# Vasodilation of rat skeletal muscle arteries by the novel BK channel opener GoSlo is mediated by the simultaneous activation of BK and K_v_7 channels

**DOI:** 10.1111/bph.14910

**Published:** 2020-01-26

**Authors:** Olga Zavaritskaya, Srikanth Dudem, Dongyu Ma, Kaneez E. Rabab, Sarah Albrecht, Dmitry Tsvetkov, Mario Kassmann, Keith Thornbury, Mitko Mladenov, Claire Kammermeier, Gerard Sergeant, Nicholas Mullins, Ornella Wouappi, Hannah Wurm, Aimo Kannt, Maik Gollasch, Mark A. Hollywood, Rudolf Schubert

**Affiliations:** ^1^ Centre for Biomedicine and Medical Technology Mannheim (CBTM), Research Division Cardiovascular Physiology, Medical Faculty Mannheim Heidelberg University Mannheim Germany; ^2^ Smooth Muscle Research Centre Dundalk Institute of Technology Dundalk Ireland; ^3^ Ion Channel Biotechnology Centre Dundalk Institute of Technology Dundalk Ireland; ^4^ Sanofi Diabetes Research Industriepark Hoechst Frankfurt am Main Germany; ^5^ Institute of Experimental and Clinical Pharmacology and Toxicology, Medical Faculty Mannheim Heidelberg University Mannheim Germany; ^6^ Department of Cardiology The First Affiliated Hospital of Anhui Medical University Anhui China; ^7^ Experimental and Clinical Research Center (ECRC), a joint cooperation between the Charité Medical Faculty and the Max Delbrück Center for Molecular Medicine (MDC) Berlin Germany; ^8^ Institute of Biology, Faculty of Natural Sciences and Mathematics, Sts. Cyril and Methodius University of Skopje Skopje Macedonia; ^9^ Department of Fundamental and Applied Physiology Russian National Research Medical University Moscow Russia; ^10^ Faculty of Medicine, Department of Physiology Augsburg University Augsburg Germany

## Abstract

**Background and Purpose:**

BK channels play important roles in various physiological and pathophysiological processes and thus have been the target of several drug development programmes focused on creating new efficacious BK channel openers, such as the GoSlo‐SR compounds. However, the effect of GoSlo‐SR compounds on vascular smooth muscle has not been studied. Therefore, we tested the hypothesis that GoSlo‐SR compounds dilate arteries exclusively by activating BK channels.

**Experimental Approach:**

Experiments were performed on rat *Gracilis* muscle, saphenous, mesenteric and tail arteries using isobaric and isometric myography, sharp microelectrodes, digital droplet PCR and the patch‐clamp technique.

**Key Results:**

GoSlo‐SR compounds dilated isobaric and relaxed and hyperpolarised isometric vessel preparations and their effects were abolished after (a) functionally eliminating K^+^ channels by pre‐constriction with 50 mM KCl or (b) blocking all K^+^ channels known to be expressed in vascular smooth muscle. However, these effects were not blocked when BK channels were inhibited. Surprisingly, the K_v_7 channel inhibitor XE991 reduced their effects considerably, but neither K_v_1 nor K_v_2 channel blockers altered the inhibitory effects of GoSlo‐SR. However, the combined blockade of BK and K_v_7 channels abolished the GoSlo‐SR‐induced relaxation. GoSlo‐SR compounds also activated K_v_7.4 and K_v_7.5 channels expressed in HEK 293 cells.

**Conclusion and Implications:**

This study shows that GoSlo‐SR compounds are effective relaxants in vascular smooth muscle and mediate their effects by a combined activation of BK and K_v_7.4/K_v_7.5 channels. Activation of K_v_1, K_v_2 or K_v_7.1 channels or other vasodilator pathways seems not to be involved.

**Abbreviations**
BK channel: calcium‐activated potassium channel of high conductance
GoSlo‐SR‐5‐130: 9,10‐dioxo‐4‐((3‐(trifluoromethyl)phenyl)amino)‐9,10‐dihydroanthracene‐2‐sulfonic acid
GoSlo‐SR‐5‐6: sodium 1‐amino‐4‐((3‐trifluoromethylphenyl)amino)‐9,10‐dioxo‐9,10‐dihydroanthracene‐2‐sulfonate
K_v_1 channel: voltage‐gated potassium channel subfamily 1
K_v_2 channel: voltage‐gated potassium channel subfamily 2
K_v_7 channel: voltage‐gated potassium channel subfamily 7
MX: methoxamine

**What is already known**

- BK channels play important roles in various physiological and pathophysiological processes.
- Several drug programmes are focused on creating new efficacious BK channel openers (e.g. GoSlo‐SR compounds).

**What this study adds**

- GoSlo‐SR compounds are effective relaxants in vascular smooth muscle.
- They mediate their effects by a combined activation of BK and K_v_7.4/K_v_7.5 channels.

**What is the clinical significance**

- GoSlo‐SR compounds may be beneficial against combined BK and K_v_7 channel dysfunction (e.g, in hypertension).

## 1 INTRODUCTION

Large conductance, calcium‐activated potassium channels (https://rgd.mcw.edu/rgdweb/report/gene/main.html?id=620715 or K_Ca_ 1.1 channels) are expressed in all tissues and organs. They contribute to a wide array of physiological functions in the kidney and neurons (Latorre et al., 2017), as well as in the heart (Balderas, Zhang, Stefani, & Toro, 2015) and both vascular (Brayden & Nelson, 1992) and visceral smooth muscle (Burdyga & Wray, 2005). Altered BK channel function has been suggested to contribute to a variety of disease states including hypertension (see discussion in Kyle & Braun, 2014), diabetes (Lu et al., 2005; McGahon et al., 2007) and detrusor overactivity (Chang et al., 2010). Thus, BK channels play important roles in various physiological processes and changes in their function may contribute to pathophysiological states.

Given these important roles, BK channels have been targeted in a number of drug development programmes (reviewed in Kaczorowski & Garcia, 2016). Many different BK channel openers have been discovered (dehydrosoyasaponin 1, McManus et al., 1993; Maxikdiol, Singh et al., 1994; DiBAC_4_, Morimoto et al., 2007) or synthesised including https://www.guidetopharmacology.org/GRAC/LigandDisplayForward?ligandId=4272 (Holland, Langton, Standen, & Boyle, 1996; Olesen, Munch, Moldt, & Drejer, 1994), the pimaranes (Imaizumi et al., 2002), NS11021 (Bentzen et al., 2007) and NS19504 (Nausch et al., 2014). Recently, a more efficacious family of BK channel openers called the GoSlo‐SR compounds have been developed (Roy et al., 2012; Roy et al., 2014). The efficacy of some of them has been reported to depend on the presence of BK channel regulatory β and γ‐subunits (Kshatri et al., 2017; Large et al., 2015; Webb et al., 2015). These compounds, in particular GoSlo‐SR‐5‐130 and GoSlo‐SR‐5‐6,activated BK channels in freshly isolated smooth muscle cells from rabbit bladder (Large et al., 2015), rabbit corpus cavernosum (Hannigan et al., 2016), and bronchial smooth muscle (Bradley et al., 2018). Furthermore, Webb et al. (2015) demonstrated that the effects of GoSlo‐SR‐5‐6 were reduced by >80%, when a triplet of mutations were introduced on the S4/S5 linker and S6 helix.

Although GoSlo‐SR compounds reliably activate BK channels in electrophysiological experiments, their effects on the contractility of intact smooth muscle tissues appear variable. Thus, Large et al. (2015) showed that GoSlo‐SR‐5‐130 decreased rabbit bladder spontaneous contractility but did not alter contractions in response to electrical field stimulation or carbachol application. In contrast, the closely related compound, GoSlo‐SR‐5‐6 failed to alter bladder contractility (Large et al., 2015). In rabbit corpus cavernosum, GoSlo‐SR‐5‐130 decreased spontaneous contractility and its effects (like those on the rabbit bladder) were reversed by https://www.guidetopharmacology.org/GRAC/LigandDisplayForward?ligandId=4218 (Hannigan et al., 2016), suggesting that these compounds mediate their effects exclusively by activating BK channels.

Even though the effects of GoSlo‐SR compounds have been established on urogenital and airways smooth muscle, little is known about their effect on vascular smooth muscle, or if these compounds open other K channels. Given that the contractility of vascular smooth muscle is modulated by a variety of K channels including BK channels (Tykocki, Boerman, & Jackson, 2017), we tested the hypothesis that GoSlo‐SR compounds dilate rat arteries exclusively by activating BK channels.

## 2 METHODS

### 2.1 Animals

The investigation conforms with the U.S. Guide for the Care and Use of Laboratory Animals (Eighth Edition, National Academy of Sciences, 2011). Animal studies are reported in compliance with the ARRIVE guidelines (McGrath & Lilley, 2015) and with the recommendations made by the *British Journal of Pharmacology.* Approval for the use of laboratory animals in this study was granted by a governmental committee on animal welfare (I‐17/17). Adult, 8‐ to 12‐week‐old, male Wistar rats were obtained from Janvier (France; RRID:RGD_13508588). Rats have been used for studies on K^+^ channel function for many years (Tykocki, Boerman, & Jackson, 2017). The animals were provided with food and water ad libitum and housed in a room with a controlled temperature and a 12‐hr light–dark cycle in IVC cages.

### 2.2 Vessel preparation

The rats were killed under CO_2_ narcosis by decapitation. The lower extremity (limb), the tail and the mesentery were quickly removed and placed in an ice‐cold physiological saline solution composed of (in mM) 145 NaCl, 4.5 KCl, 1.2 NaH_2_PO_4_, 0.1 CaCl_2_, 1.0 MgSO_4_, 0.025 EDTA, 5 HEPES at pH 7.4. All arteries were isolated by removing all surrounding skeletal muscle and connective tissue. Small rings 2 mm in length were used for further experiments.

### 2.3 Isobaric mounting of *Gracilis* arteries

Vessels were mounted on two glass pipettes in the experimental chamber of an isobaric myograph (201CM, Danish Myotechnology, Denmark) containing experimental solution (physiological saline solution, PSS) consisting of (in mM) 146 NaCl, 4.5 KCl, 1.2 NaH_2_PO_4_, 1.0 MgSO_4_, 1.6 CaCl_2_, 0.025 EDTA, 5.5 glucose, and 5 HEPES at pH 7.4. The microscope image of the vessel was viewed with a CCD camera and digitised by a frame‐grabber card (Hasotec, Gemany). Based on the vessel image, diameter changes were measured continuously at a sampling rate of 0.5 Hz using a custom‐made programme (Fischer, Mewes, Hopp, & Schubert, 1996). Vessels were exposed to a pressure of 80 mmHg without any luminal flow at a temperature of 37°C. To ensure complete non‐flow conditions, leaking vessels were discarded at any stage of the experiment. After development of a spontaneous myogenic tone, vessel viability was tested with https://www.guidetopharmacology.org/GRAC/LigandDisplayForward?ligandId=484 at 10^−5^ M to test smooth muscle cell function and https://www.guidetopharmacology.org/GRAC/LigandDisplayForward?ligandId=294 (ACh) at 10^−6^ M to test endothelial cell function. At the end of the experiments, all vessels were exposed to calcium‐free solution to determine the fully relaxed diameter at 80 mmHg. The fully relaxed diameter of the vessels in this study was in the range from 240 to 368 μm. All diameter values were normalised to the diameter of the fully relaxed vessel at 80 mmHg in a calcium‐free solution. Normalisation was done in order to eliminate variability due to differences in the size of different vessels.

### 2.4 Isometric mounting of arteries

Isolated vessels were mounted in a wire myograph (model 410A or 610M, Danish Myotechnology, Denmark) for recording of isometric tension on two wires with a diameter of 40 μm. Data acquisition and analysis was performed using Labchart (ADInstruments, USA). The arteries were stretched to their optimal lumen diameter (90% of the diameter they would have at a transmural pressure of 100 mmHg; Mulvany & Halpern, 1977; wall tension under these conditions corresponds to a pressure of about 45 mmHg according to the law of Laplace) and studied in PSS consisting of (in mM) 120 NaCl, 4.5 KCl, 1.2 NaH_2_PO_4_, 1.0 MgSO_4_, 1.6 CaCl_2_, 0.025 EDTA, 5.5 glucose, 26 NaHCO_3_, and 5 HEPES at pH 7.4 oxygenated with carbogen (95% O_2_ and 5% CO_2_) at 37°C. Viability of the vessels was tested with https://www.guidetopharmacology.org/GRAC/LigandDisplayForward?ligandId=483 (MX) at 10^−5^ M to test smooth muscle cell function and ACh at 10^−5^ M after pre‐constriction with 10^−7^ M methoxamine to test endothelial cell function. The solution containing 50 mM KCl was prepared based on PSS by equimolar replacement of NaCl. Vessel tension was normalised to the peak tension developed in response to 10^−5^ M methoxamine applied directly after the viability test in order to eliminate variability due to differences in the contractility of different vessels. To be able to compare vessel responses to different interventions, special care was taken to carefully match vessel tension before the intervention. For example, pre‐constrictions obtained (a) before application of the GoSlo‐SR compound by application of methoxamine alone in the control group of vessels and (b) by application of methoxamine together with IBTX in the treatment group of vessels (different vessels compared to the control group) were the same (see Figure 5b, time point “0”).

### 2.5 Functional removal of the endothelium

In the experiments of this study, the endothelium of the vessels was removed. In isobaric experiments, this was done by passing an air bubble through the lumen of the vessel. In isometric experiments, mechanical disruption of endothelium using a rat whisker was performed. Functional removal of the endothelium was considered successful when ACh‐induced vasodilation was absent during the viability test.

### 2.6 Membrane potential recordings

Intracellular recordings of membrane potential in smooth muscle cells of intact mesenteric arteries were made using microelectrodes pulled from aluminosilicate glass and filled with 3 M KCl. An amplifier (DUO 773, World Precision Instruments) was used to record the membrane potential. A micromanipulator (UMP, Sensapex) was employed to make impalements from the adventitial side of the vessel. The following criteria for acceptance of membrane potential recordings were used: (a) an abrupt change in membrane potential upon cell penetration; (b) a constant electrode resistance when compared before, during, and after the measurement; (c) a stable reading of the membrane potential lasting longer than 1 min; (d) no change in the baseline when the electrode was removed.

### 2.7 Digital droplet PCR

Vessels, isolated as described above, were cut into small pieces and homogenised for 3 min at 30 Hz in the TissueLyser (Qiagen). Total RNA was isolated using the “miRNeasy Mini‐Kit” (Qiagen) according to the manufacturer instructions. Optional On‐Column DNase Digestion using the RNase‐Free DNase Set (Qiagen) was performed as described. In the final step, RNA was collected from the affinity column using 30 μl H_2_O, which was passed twice over the column. RNA concentration was determined on the Tecan infinite 200.

Samples were quantified by two‐step digital droplet PCR. Reverse transcription to cDNA was done using the iScript™ cDNA Synthesis Kit (Bio‐Rad, Hercules, CA, Cat#170‐8890) according to the manufacturer's standard protocol. All samples were diluted to a starting concentration of 12.5 ng RNA per microliter of reaction.

Primers and probes were either purchased from Bio‐Rad or self‐designed and ordered from Sigma‐Aldrich (St. Louis, MO). All probes were FAM‐labelled at the 5′‐end, except GAPDH which was HEX‐labelled, and BHQ1‐labelled at the 3′‐end. Amplicon context sequence and amplicon length can be found on the Bio‐Rad Homepage (http://www.bio-rad.com) in accordance with the Guidelines for Minimum Information for Publication of Quantitative Digital PCR Experiments (MIQE; Huggett et al., 2013). The following genes (with their Unique Assay ID or Sequence) have been tested: KCNMA1 (dRnoCPE5151992), KCNQ1 (dRnoCPE5168228), KCNQ2 (dRnoCPE5150290), KCNQ3 (dRnoCPE5174006), KCNQ4 (dRnoCPE5184994), KCNQ5 (ffw: TGTACAACGTGCTGGAGAGAC, rev: ACGATCATCACGAACTCCAGAA, Prb: CCCGCGGCTGGGCGTTCGTCT), and GAPDH (dRnoCPE5188005). The annealing temperature was set to 58.0°C based on a temperature gradient run. The limit of detection, the linearity of amplification, and the possibility to do duplex measurements were checked by dilution of synthetic oligonucleotides corresponding to the specific probe sequences. All primers and probes were used at a concentration of 900 and 250 nM, respectively.

Samples were quantified with digital droplet PCR (Hindson et al., 2011) using the ddPCR™ Supermix for Probes (no dUTP; Bio‐Rad, Cat#186‐3024) on a QX200™ AutoDG™ Droplet Digital™ PCR System (Bio‐Rad) according to the manufacturer's standard protocol. For each reaction of 20 μl, a volume of 1 μl cDNA was used (as an equivalent of 12.5 ng starting RNA). Typically, measurements were repeated until a minimum of 100 positive droplets were detected (between two and nine independent experiments). For KCNQ2, expression was too low in the limited amount of material to observe 100 positive droplets. Each experiment included a negative control (no template control) and a positive control (synthetic oligo corresponding to the probe). The QuantaSoft analysis software (Version 1.7, Bio‐Rad) was used to analyse the ddPCR data.

### 2.8 Cell isolation

A piece of a tail or mesenteric artery was placed into a microtube containing 1 ml of an isolation solution consisting of (in mM) 55 NaCl, 6 KCl, 88 Na glutamate, 2 MgCl_2_, 10 HEPES, 10 glucose, pH 7.4, as well as 0.6 mg·ml^−1^ https://www.guidetopharmacology.org/GRAC/FamilyDisplayForward?familyId=728, and 1.2 mg·ml^−1^ DL‐DTT for 20 min at 37°C. Thereafter, the artery was moved into a microtube containing 1 ml of the isolation solution as well as 1.2 mg·ml^−1^ collagenase F, 1.0 mg·ml^−1^ trypsin inhibitor, and 0.5 mg·ml^−1^ elastase for 12 min at 37°C for cells from tail arteries or in isolation solution containing 1 mg·ml^−1^ collagenase (types F and H; ratio, 30% and 70%, respectively) and 0.1 mM CaCl_2_ for 16 min at 37°C for cells from mesenteric arteries. Single cells were released by trituration with a polyethylene pipette into the experimental bath solution consisting of (in mM) 126 NaCl, 4.5 KCl, 1 MgCl_2_, 0.1 CaCl_2_, 10 HEPES, 20 taurine, 20 glucose and 5 pyruvate at pH 7.4. The pipette solution contained (in mM) 109 KCl, 10 NaCl, 1 MgCl_2_, 2 CaCl_2_, 3 EGTA (purity 96%) and 10 HEPES.

### 2.9 Patch‐clamp recording on freshly isolated cells

All experiments were performed in the whole cell mode at room temperature. Patch pipettes had resistances of 2–5 MΩ. The recordings were made with an Axopatch 200B amplifier. Stimulation of currents and data analysis were done with the software package ISO2 or with pClamp software version 10.2 (RRID:SCR_011323). BK currents were isolated from K_v_ currents using a depolarised holding potential of 0 mV eliminating all inactivating currents. Initial experiments had shown that neither 1 μM https://www.guidetopharmacology.org/GRAC/LigandDisplayForward?ligandId=2414, an inhibitor of ATP‐sensitive potassium channels, nor 10 μM barium, an inhibitor of inward‐rectifying potassium channels, produced any effect on the outward current of these cells. BK currents were normalised to the BK current evoked at 50 mV immediately before the recording of the control current–voltage relationship in order to account for the different size of the BK currents in different cells.

### 2.10 Cell culture and transfection

HEK 293 cells (RRID:CVCL_0045) were cultured in DMEM media containing 10% FBS and 1% penicillin, streptomycin antibiotics at 37°C in a humidifying incubator with 5% CO_2_. Subculturing was done with a 0.5% Trypsin–EDTA solution. The day before transfection, the cells were plated in 35 mm dishes. cDNA complexes were diluted in 100 μl serum‐free media tube. In another tube, 3 μl lipofectamine reagent was diluted in 100 μl serum‐free media. These two solutions were mixed together and incubated for 30 min at room temperature. Immediately prior to transfection, the media in each dish were changed to a serum, and antibiotic‐free media before 200 μl of the transfection mixture were gently added to each dish in a drop wise manner. Transfection was terminated after 4 hr by replacing the media in each dish with fresh growth media.

### 2.11 Patch‐clamp recording on cultured HEK cells

Electrophysiological recordings were made on single HEK cells 24‐ to 48‐hr post‐transfection with cDNA for human K_v_7.4, K_v_7.4_W242L_, and K_v_7.5. The plasmids were kindly supplied as gifts from Prof Søren Peter Olesen, University of Copenhagen, Denmark. All experiments were carried out at room temperature. Patch pipettes were pulled from thin‐walled borosilicate glass (1.5 mm O.D. × 1.17 mm I.D.; Clark Medical Instruments) to a tip of diameter approximately 1–1.5 μm and resistance of 2–5 MΩ. Voltage clamp commands were delivered via an Axopatch 200A amplifier (Axon Instruments) connected to a Digidata 1322A AD/DA converter (Axon Instruments) interfaced to a computer running pClamp software (Axon Instruments). The data were acquired at 10 kHz and filtered at 2 kHz. Series resistance was uncompensated in these experiments, we estimate that errors resulting from this were <20 mV.

All experiments were carried out in the whole cell configuration of the patch‐clamp technique (Hamill, Marty, Neher, Sakmann, & Sigworth, 1981). Cells were held at −80 mV and stepped from −100 to +50 mV for 1 s in 10 mV increments with a 10 s interval between steps. Activation curves were constructed from the peak tail current evoked by a repolarisation back to −120 mV following depolarising voltage steps. Data were fitted with the Boltzmann equation of the form:

$$I/I_{\max}=1/\left(1+\exp\left(\left(V_{1/2}-\mathrm{Vm}\right)/K\right)\right),$$

where *V*_1/2_ was the membrane potential at which there was half maximal activation, *K* the slope factor, and *Vm* the membrane potential (mV). The change in activation *V*_1/2_ (Δ*V*_1/2_) caused by drugs was obtained by subtracting the *V*_1/2_ in control from that in the presence of the drugs. Leak current was estimated from the current at the end of the −120 mV repolarisation step in the absence of any drugs and was digitally subtracted.

During experiments, the dish containing HEK cells was superfused with Hank's solution (for composition, see next paragraph). In addition, the cell under study was continuously superfused with Hank's solution by means of a close delivery system consisting of a pipette (tip diameter 200 μm) placed approximately 300 μm away from the cell. This could be switched, with a dead‐space time of around 5 s, to a solution containing a drug.

### 2.12 Recording solutions for cultured cells

The composition of the solutions used was as follows (in mM): Hank's solution: 129.8 Na^+^, 5.8 K^+^, 135 Cl^−^, 4.17 HCO_3_^−^, 0.34 HPO_4_^2−^, 0.44 H_2_PO_4_^−^, 1.8 Ca^2+^, 0.9 Mg^2+^, 0.4 SO_4_^2−^, 10 glucose, 2.9 sucrose and 10 HEPES, pH adjusted to 7.4 with NaOH. K^+^ pipette solution (whole cell, in mM)*:* 132 K^+^, 110 gluconate, 21 Cl^−^, 2 Na^+^, 0.5 Mg^2+^, 1 ATP, 0.1 GTP, 2.5 phosphocreatine, 5 HEPES, and 1 EGTA; pH adjusted to 7.2 with KOH.

### 2.13 Materials

Methoxamine, ACh, https://www.guidetopharmacology.org/GRAC/LigandDisplayForward?ligandId=2343 and the salts for the solutions were obtained from Sigma (Germany). Iberiotoxin, penitrem A, and stromatoxin were purchased from Alomone Labs (Isreal). DPO‐1 and XE991 were obtained from Tocris (UK). GoSlo‐SR‐5‐6 (sodium 1‐Amino‐4‐((3‐trifluoromethylphenyl)amino)‐9,10‐dioxo‐9,10‐dihydroanthracene‐2‐sulfonate) was prepared as described previously (Roy et al., 2012; Roy et al., 2014) with a modified purification protocol. A suspension of bromaminic acid sodium salt (0.20 g, 0.49 × 10^−3^ M), 3‐trifluoromethyl aniline (0.12 ml, 0.99 × 10^−3^ M), and copper powder (8 mg) in a buffer solution of 0.2 M Na_2_HPO_4_ (4 ml) and 0.12 M NaH_2_PO_4_ (4 ml) was irradiated for 20 min at 110°C in a microwave oven. The reaction mixture was cooled to room temperature, filtered, and diluted with water (200 ml). The aqueous solution was extracted with dichloromethane (2–3 × 200 ml) until the organic layer became colourless indicating complete removal of unreacted amine starting material. The aqueous layer was then saturated with solid NaCl and extracted with ethyl acetate (2 × 200 ml). The combined ethyl acetate layers were then washed with 5% aqueous NaCl solution (5–6 × 200 ml) in order to remove unreacted bromaminic acid. The organic layer was then dried over anhydrous Na_2_SO_4_, filtered, and evaporated to give the title product as a blue solid (99 mg, 42%). Spectroscopic data were in agreement with that already reported.

GoSlo‐SR‐5‐130 [9,10‐dioxo‐4‐((3‐trifluoroemthyl)phenyl)amino)‐9,10,‐dihydroanthracene‐2‐sulfonic acid] was synthesised using an adapted method from that described previously (Roy et al., 2012; Roy et al., 2014). To a stirring suspension of GoSlo‐SR‐5‐6 (75 mg, 0.15 × 10^−3^ M) in 1 M HCl (10 ml) at 0°C, was added an aqueous solution of 1.5 ml of NaNO_2_ (0.60 × 10^−3^ M). The reaction mixture was stirred at 0°C for 5 min, warmed to room temperature, and allowed to stir for a further 1 hr; ethanol (10 ml) and zinc dust (65 mg, 1.5 × 10^−3^ M) were added. After 5 min, the reaction was quenched by the addition of a 0.5 M aqueous NaHCO_3_ solution and extracted with ethyl acetate (2 × 50 ml). The combined organic layers were dried over anhydrous Na_2_SO_4_, filtered, evaporated, and the residue purified by flash chromatography (methanol‐dichloromethane) to afford the title product as a purple solid (46 mg, 69%). Spectroscopic data were in agreement with that already reported.

### 2.14 Statistics

The data and statistical analysis comply with the recommendations of the *British Journal of Pharmacology* on experimental design and analysis in pharmacology (Curtis et al., 2015; Curtis et al., 2018). All values are given as mean ± SEM; *n* is the number of animals tested or the number of cells recorded from, technical replicates were not treated as independent values; groups in one experimental series were of equal size. Group size selection was based on previous extensive experience. The allocation of individual vessels to different treatments of an experimental series was randomised. Blinding of the operator was not feasible because vessel responses observed by the operator to manage the experiment permitted inferences about the treatment. However, data analysis was performed semi‐blinded by an independent analyst. Outliers were included in data analysis and presentation. Statistical analysis was performed using GraphPadPrism 6.0 (RRID:SCR_002798; GraphPad Software, Inc.) employing ANOVA (parametric test as there was no significant variance inhomogeneity; post hoc tests were conducted only if F in ANOVA achieved P < .05), unpaired or paired Student's *t‐*tests, as appropriate and only on groups with at least *n* = 5. For methodological reasons, a few groups did not reach *n* = 5, these data have not been subjected to statistical analysis. A value of *P* < .05 was considered statistically significant.

### 2.15 Nomenclature of targets and ligands

Key protein targets and ligands in this article are hyperlinked to corresponding entries in http://www.guidetopharmacology.org, the common portal for data from the IUPHAR/BPS Guide to PHARMACOLOGY (Harding et al., 2018), and are permanently archived in the Concise Guide to PHARMACOLOGY 2019/20 (Alexander et al., 2019).

## 3 RESULTS

### 3.1 Effect of GoSlo‐SR compounds on myogenic tone

**Figure 1:**
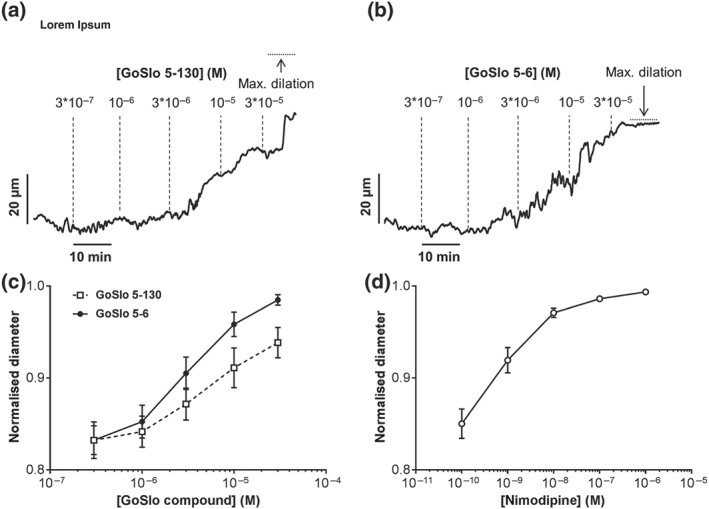
GoSlo‐SR‐5‐130 and GoSlo‐SR‐5‐6 cause concentration‐dependent relaxations of isobaric preparations of rat *Gracilis* muscle arteries. (a) Example of the effect of GoSlo‐SR‐5‐130 on the diameter of an isobaric vessel preparation at 80 mmHg, initial vessel diameter 287 μm, maximum vessel diameter 365 μm. (b) Example of the effect of GoSlo‐SR‐5‐6 on the diameter of an isobaric vessel preparation at 80 mmHg, initial vessel diameter 283 μm, maximum vessel diameter 343 μm. (c) Effect of GoSlo‐SR compounds on spontaneous tone of isobaric vessel preparations at 80 mmHg. Normalised vessel diameter (ratio of diameter/fully relaxed diameter at 80 mmHg) at different concentrations of GoSlo‐SR compounds. (GoSlo‐SR‐5‐130 one‐way ANOVA: *n* = 6; *P* < .05), (GoSlo‐SR‐5‐6 one‐way ANOVA: *n* = 7; *P* < .05; repeated measures ANOVA GoSlo‐SR‐5‐6 vs. GoSlo‐SR‐5‐130: *P* < .05). (d) Effect of nimodipine on spontaneous tone of isobaric vessel preparations at 80 mmHg. Normalised vessel diameter (ratio of diameter/fully relaxed diameter at 80 mmHg) at different concentrations of nimodipine (one‐way ANOVA: *n* = 12; *P* < .05)

Isobaric preparations of rat *Gracilis* muscle arteries possessing spontaneous myogenic tone at 80 mmHg were dilated in a concentration‐dependent manner by both GoSlo‐SR‐5‐130 (Figure 1a,c) and GoSlo‐SR‐5‐6 (Figure 1b,c). The L‐type calcium‐channel antagonist https://www.guidetopharmacology.org/GRAC/LigandDisplayForward?ligandId=2523 also produced dilation initiated, however, at lower concentrations (Figure 1d).

**Figure 2:**
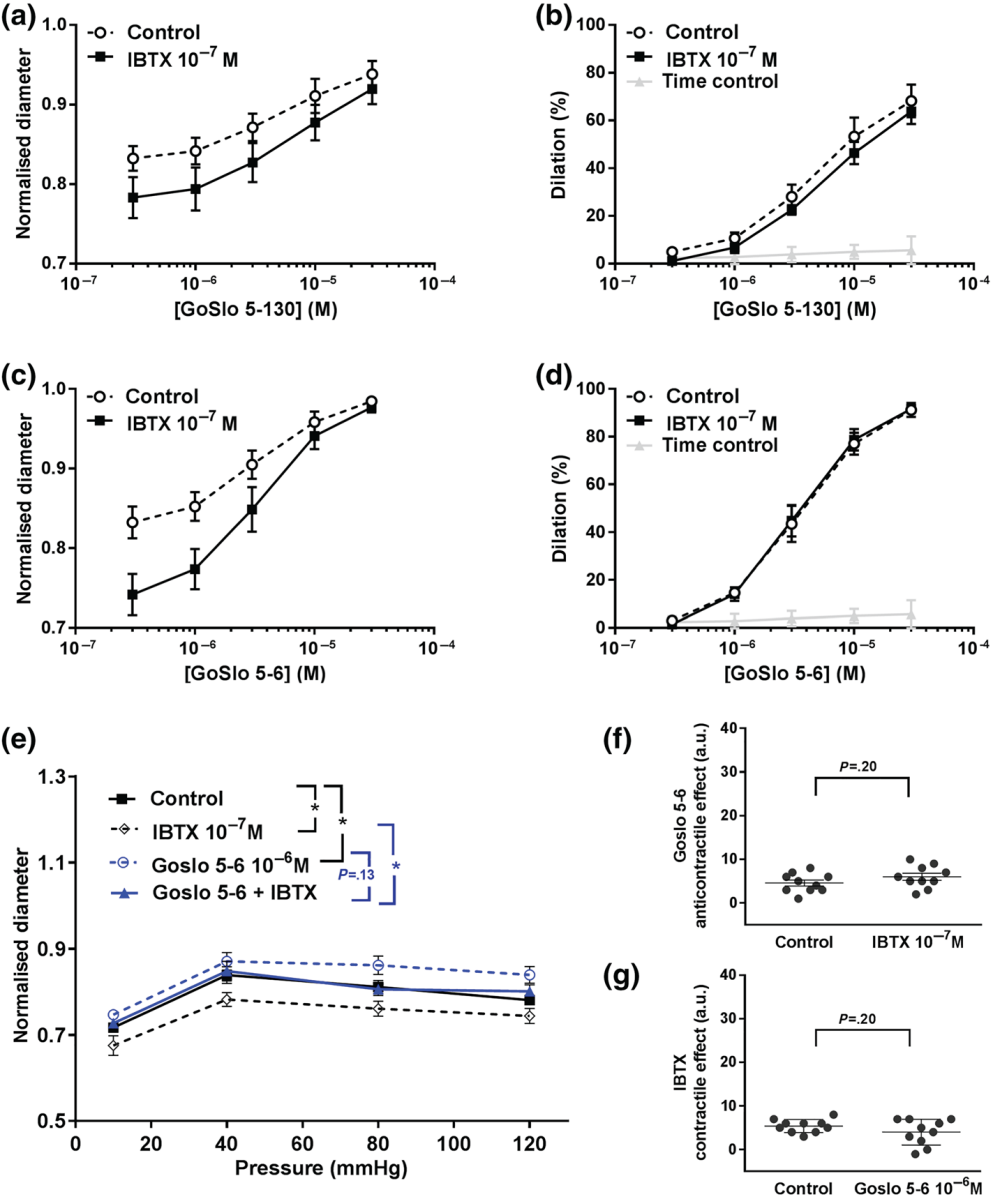
The relaxant effects of GoSlo‐SR compounds are not abolished when BK channels are blocked. (a) Effect of GoSlo‐SR‐5‐130 on spontaneous tone of isobaric vessel preparations at 80 mmHg. Normalised vessel diameter (ratio of diameter/fully relaxed diameter at 80 mmHg) at different concentrations of GoSlo‐SR‐5‐130 in the absence (Control) and presence of 10^−7^ M IBTX (IBTX; repeated measures ANOVA: *n* = 6; *P* = 0.20); (b) vessel dilation in the absence (Time control = application of GoSlo‐SR solvent DMSO) and presence of GoSlo‐SR‐5‐130 (Control; repeated measures ANOVA: *n* = 6; *P* < .05), vessel dilation at different concentrations of GoSlo‐SR‐5‐130 in the absence (Control) and presence of 10^−7^ M IBTX (IBTX; repeated measures ANOVA: *n* = 6; *P* = 0.28); (c) effect of GoSlo‐SR‐5‐6 on spontaneous tone of isobaric vessel preparations at 80 mmHg. Normalised vessel diameter (ratio of diameter/fully relaxed diameter at 80 mmHg) at different concentrations of GoSlo‐SR‐5‐6 in the absence (Control) and presence of 10^−7^ M IBTX (IBTX; repeated measures ANOVA: *n* = 7; *P* < .05); (d) vessel dilation in the absence (Time control = application of GoSlo‐SR solvent DMSO) and presence of GoSlo‐SR‐5‐6 (Control; repeated measures ANOVA: *n* = 7; *P* < .05), vessel dilation at different concentrations of GoSlo‐SR‐5‐6 in the absence (Control) and presence of 10^−7^ M IBTX (IBTX; repeated measures ANOVA: *n* = 7; *P* = 0.94); (e) effect of GoSlo‐SR‐5‐6 on the pressure–diameter relationship of isobaric vessel preparations. Normalised vessel diameter (ratio of diameter/fully relaxed diameter at 80 mmHg) at different pressures in the absence of any substances (Control), in the presence of 10^−6^ M GoSlo‐SR‐5‐6, in the presence of 10^−7^ M IBTX and in the presence of both GoSlo‐SR‐5‐6 and IBTX (repeated measures ANOVA: *n* = 10; ^*^ *P* < .05); (f) effect of GoSlo‐SR‐5‐6 in the absence (Control) and presence of 10^−7^ M IBTX (unpaired Student's *t‐* test: *n* = 10). The effect of GoSlo‐SR‐5‐6 in the absence of IBTX was quantified as (area under the GoSlo‐SR‐5‐6 curve) − (area under the control curve). The effect of GoSlo‐SR‐5‐6 in the presence of IBTX was quantified as (area under the GoSlo‐SR‐5‐6 + IBTX curve) − (area under the IBTX curve); (g) effect of IBTX in the absence (Control) and presence of 10^−6^ M GoSlo‐SR‐5‐6 (unpaired Student's *t‐*test: *n* = 10). The effect of IBTX in the absence of GoSlo‐SR‐5‐6 was quantified as (area under the control curve) − (area under the IBTX curve). The effect of IBTX in the presence of GoSlo‐SR‐5‐6 was quantified as (area under the GoSlo‐SR‐5‐6 curve) − (area under the GoSlo‐SR‐5‐6 + IBTX curve)

Recent publications (Hannigan et al., 2016; Large et al., 2015) suggest that the inhibitory effects of the GoSlo‐SR family of compounds are mediated via activation of BK channels. Therefore, we were surprised to find that inhibition of BK channels with IBTX (10^−7^ M; Galvez et al., 1990) failed to reduce the effect of either GoSlo‐SR‐5‐130 (Figure 2a,b) or GoSlo‐SR‐5‐6 (Figure 2c,d) on spontaneous myogenic tone in isobaric preparations of rat *Gracilis* muscle arteries studied at 80 mmHg. However, it is important to note that BK channels are functionally expressed in this preparation since application of IBTX clearly contracted these vessels in experiments subsequently testing GoSlo‐SR‐5‐130 and in experiments subsequently testing GoSlo‐SR‐5‐6. Furthermore, GoSlo‐SR‐5‐6 shifted the pressure–diameter relationship of these vessels to larger diameters, an effect which was not blocked by IBTX (Figure 2e–g).

**Figure 3:**
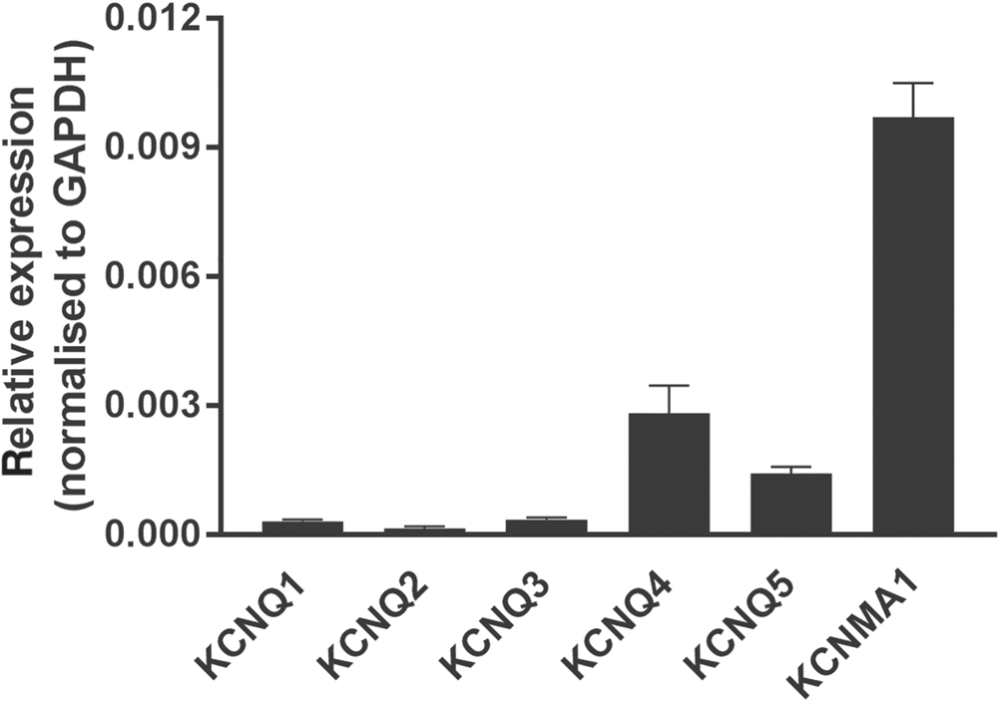
mRNA expression of BK and K_v_7 channels. Relative expression of BK and KCNQ channels in intact arteries normalised to GAPDH

These data suggest that GoSlo‐SR compounds dilate arteries even when BK channels are blocked. As we show later, their effect is abolished in high [K^+^]_o_ solutions, supporting the idea that they mediate their effects via activation of K^+^ channels. There is another functionally important class of K^+^ channels, the K_v_7 channels, widely expressed in vascular smooth muscle (see recent reviews of Barrese, Stott, & Greenwood, 2018; Byron & Brueggemann, 2018; Haick & Byron, 2016). Thus, we first quantified transcriptional expression of KCNQ1–KCNQ5 using digital PCR and compared it to KCNMA1 as shown in Figure 3. As this figure suggests, only https://rgd.mcw.edu/rgdweb/report/gene/main.html?id=61799 and https://rgd.mcw.edu/rgdweb/report/gene/main.html?id=628848 mRNA levels were abundantly expressed, relative to KCNMA1, in *Gracilis* arteries.

**Figure 4:**
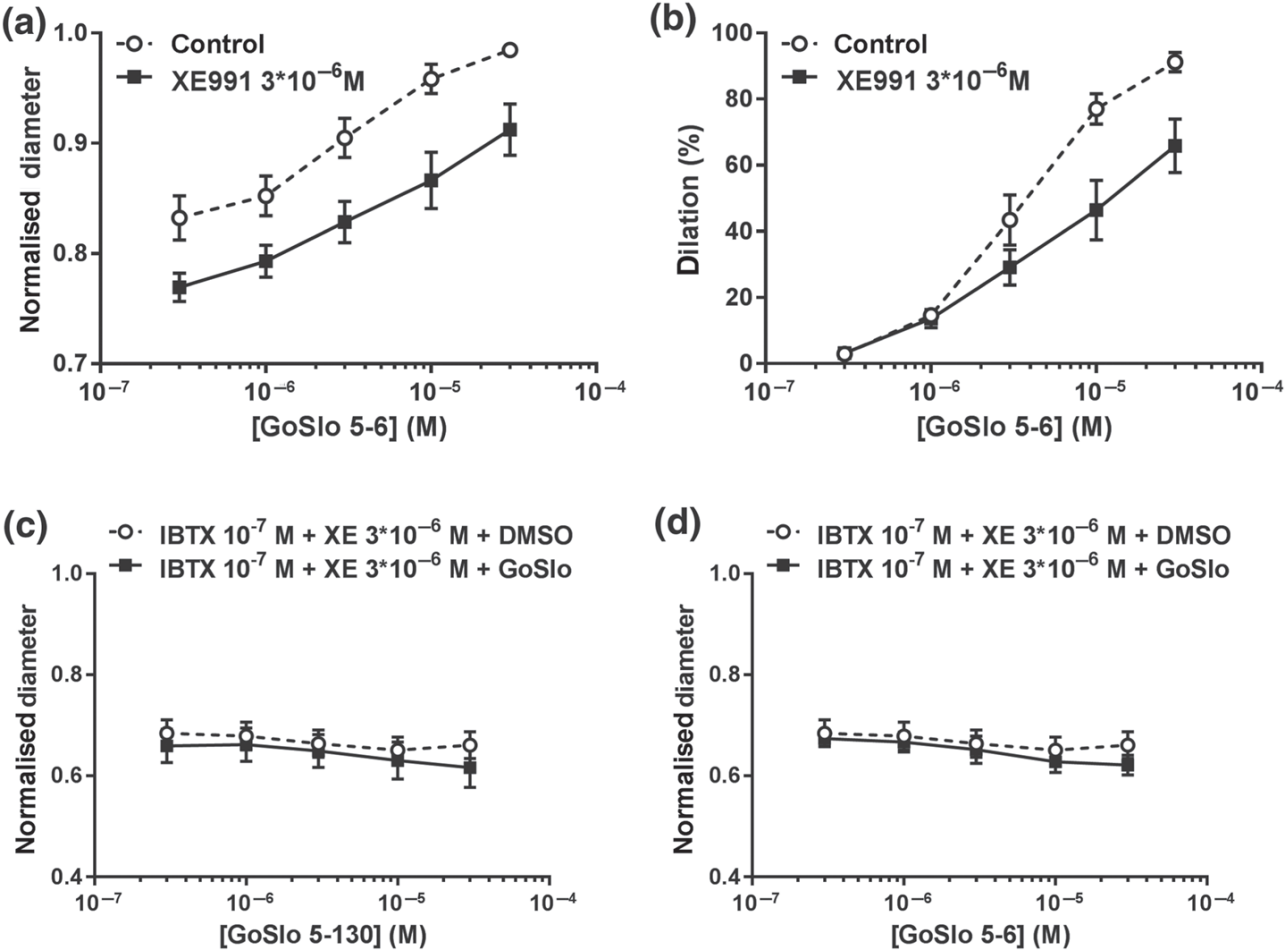
Effects of GoSlo‐SR compounds are reduced when K_v_7 channels are blocked and abolished when both BK and K_v_7 channels are inhibited. (a) Effect of GoSlo‐SR‐5‐6 on spontaneous tone of isobaric vessel preparations at 80 mmHg. Normalised vessel diameter (ratio of diameter/fully relaxed diameter at 80 mmHg) at different concentrations of GoSlo‐SR‐5‐6 in the absence (Control) and presence of 3 × 10^−6^ M XE991 (XE991; repeated measures ANOVA: *n* = 10; *P* < .05); (b) vessel dilation at different concentrations of GoSlo‐SR‐5‐6 in the absence (Control) and presence of 3 × 10^−6^ M XE991 (XE991; repeated measures ANOVA: *n* = 10; *P* < .05); (c) effect of GoSlo‐SR‐5‐130 on spontaneous tone of isobaric vessel preparations at 80 mmHg. Normalised vessel diameter (ratio of diameter/fully relaxed diameter at 80 mmHg) at different concentrations of GoSlo‐SR‐5‐130 in the presence of 10^−7^ M IBTX and 3 × 10^−6^ M XE991 in the absence (DMSO) and presence of GoSlo‐SR‐5‐130 (GoSlo; repeated measures ANOVA: *n* = 6; *P* = .59); (d) effect of GoSlo‐SR‐5‐6 on spontaneous tone of isobaric vessel preparations at 80 mmHg. Normalised vessel diameter (ratio of diameter/fully relaxed diameter at 80 mmHg) at different concentrations of GoSlo‐SR‐5‐6 in the presence of 10^−7^ M IBTX and 3 × 10^−6^ M XE991 in the absence (DMSO) and presence of GoSlo‐SR‐5‐6 (GoSlo; repeated measures ANOVA: *n* = 6; *P* = .56)

Having established the transcriptional expression of KCNQ, we next examined the effects of a K_v_7 channel blocker, https://www.guidetopharmacology.org/GRAC/LigandDisplayForward?ligandId=2596 (3 × 10^−6^ M; Greenwood & Ohya, 2009) on isobaric preparations pressurised to 80 mmHg. As shown in Figure 4a,b, GoSlo‐SR‐5‐6 produced a concentration‐dependent dilation of these arteries and this effect was reduced but not abolished when K_v_7 channels were blocked with XE991. Of note, XE991 significantly contracted these vessels by 6.4 ± 1.1% which was not different from the effect of IBTX as reported above. Interestingly, this effect of GoSlo‐SR‐5‐6 was unaltered by the K_v_7.1 channel blocker HMR1556 (10^−5^ M, *n* = 5), suggesting that either GoSlo‐SR‐5‐6 did not mediate its effects by activating K_v_7.1 channels or K_v_7.1 channels are not functionally expressed in these vessels.

We next examined if a combination of blocking BK channels with IBTX (10^−7^ M) and K_v_7 channels with XE991 (3 × 10^−6^ M) could further reduce the effects of the GoSlo‐SR compounds on isobaric preparations. Blockade of both channels abolished the relaxant effects of GoSlo‐SR‐5‐130 (Figure 4c) and GoSlo‐SR‐5‐6 (Figure 4d) on myogenic tone, at all concentrations tested.

### 3.2 Effect of GoSlo‐SR compounds on agonist‐induced tone

**Figure 5:**
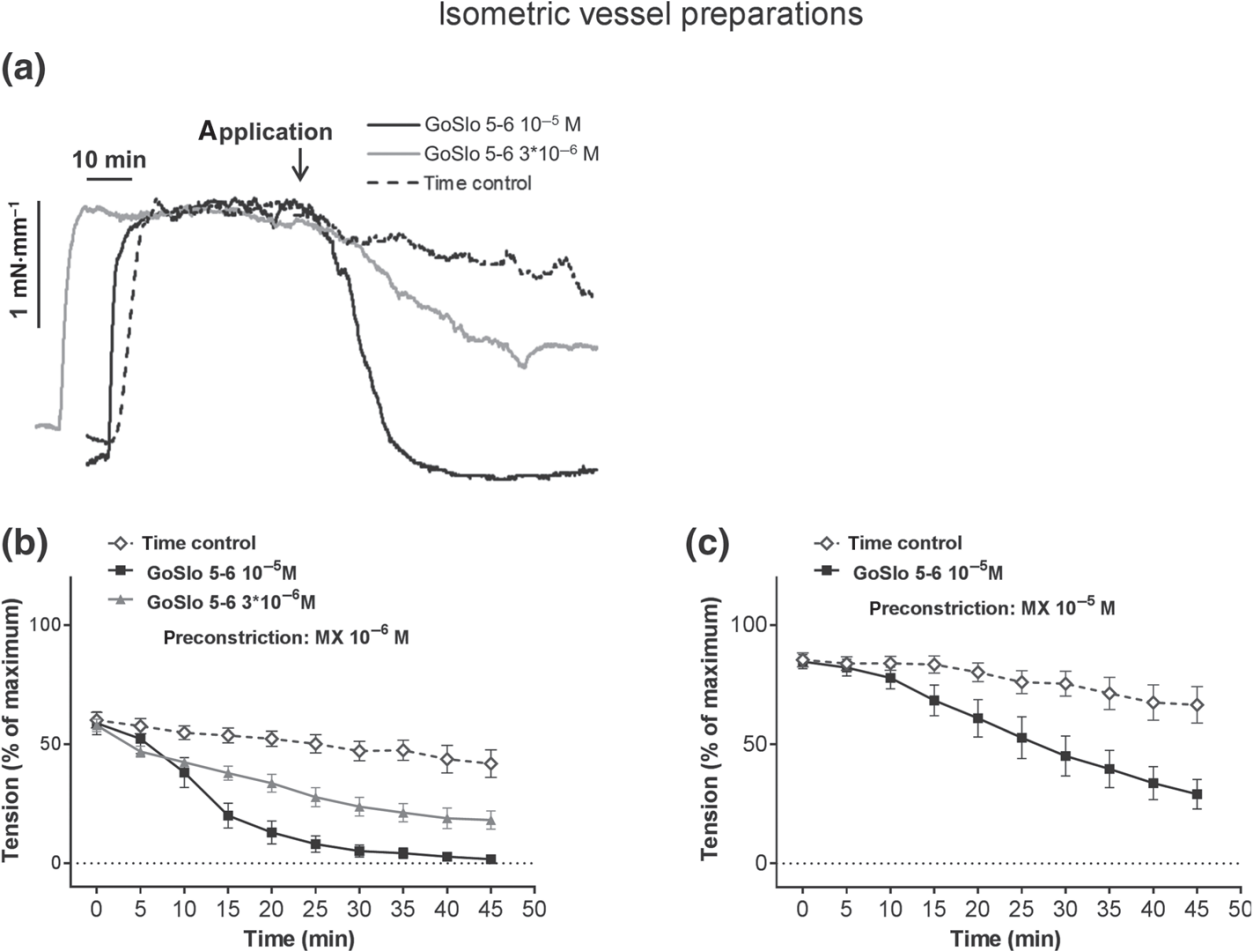
GoSlo‐SR‐5‐6 also causes concentration‐dependent relaxations of isometric preparations of rat *Gracilis* muscle arteries. (a) Example of the effect of GoSlo‐SR‐5‐6 on tension of an isometric vessel preparation at 10^−6^ M methoxamine (MX)‐induced tone. Application denotes the time point where GoSlo‐SR or vehicle was added. (b) Effect of GoSlo‐SR‐5‐6 on 10^−6^ M MX‐induced contraction. Vessel tension in the absence (Time control) and presence of GoSlo‐SR‐5‐6 at 3 × 10^−6^ M and at 10^−5^ M (repeated measures ANOVA: con vs. GoSlo‐SR‐5‐6 10^−5^ M: *n* = 11; *P* < .05; con vs. GoSlo‐SR‐5‐6 3 × 10^−6^ M: *n* = 8; *P* < .05; GoSlo‐SR‐5‐6 10^−5^ M vs. 3 × 10^−6^ M: *P* < .05); (c) effect of GoSlo‐SR‐5‐6 on 10^−5^ M MX‐induced contraction. Vessel tension in the absence (Time control) and presence of GoSlo‐SR‐5‐6 at 10^−5^ M (repeated measures ANOVA: *n* = 8; *P* < .05)

To test if GoSlo‐SR compounds mediated a relaxant effect on agonist‐induced isometric tone by activating both BK and K_v_7 channels, we next examined the effects of GoSlo‐SR‐5‐6 on vessels preconstricted with the α_1_ adrenoceptor agonist, methoxamine (MX, 10^−6^ M). As Figure 5a,b suggests, application of 3 × 10^−6^ M and 10^−5^ M GoSlo‐SR‐5‐6 caused concentration‐dependent relaxations. Interestingly, GoSlo‐SR‐5‐6 was less effective at relaxing tone induced with a higher concentration (10^−5^ M) of MX (Figure 5c).

**Figure 6:**
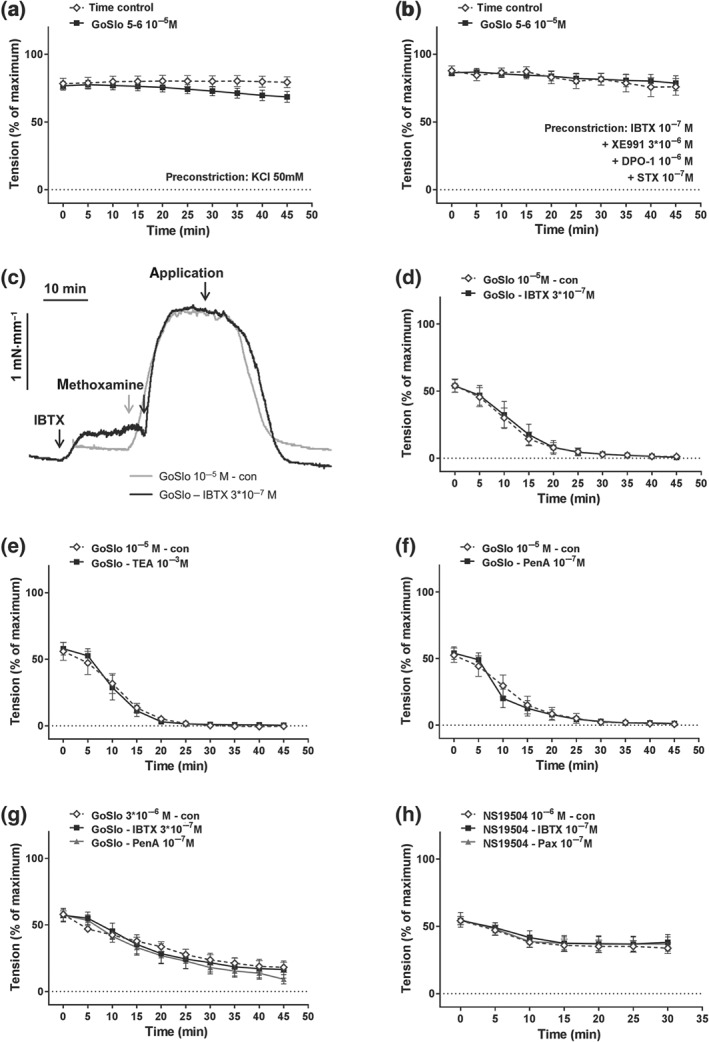
GoSlo‐SR‐5‐6‐induced relaxations of isometric tone depend on K^+^ channels but are not abolished when BK channels are blocked. (a) Effect of GoSlo‐SR‐5‐6 on 50 mM KCl‐induced contraction. Vessel tension in the absence (Time control) and presence of GoSlo‐SR‐5‐6 at 10^−5^ M (repeated measures ANOVA: *n* = 10; *P* = .29); (b) effect of GoSlo‐SR‐5‐6 on contraction induced by 10^−7^ M IBTX, 3 × 10^−6^ M XE991, 10^−6^ M DPO‐1 and 10^−7^ M stromatoxin. Vessel tension in the absence (Time control) and presence of GoSlo‐SR‐5‐6 at 10^−5^ M (repeated measures ANOVA: *n* = 6; *P* = .88); (c) example of the effect of IBTX on relaxation induced by 10^−5^ M GoSlo‐SR‐5‐6. Application denotes the time point where GoSlo‐SR was added. (d) Effect of 10^−5^ M GoSlo‐SR‐5‐6 on methoxamine (MX)‐induced contraction. Vessel tension in the absence (con) and presence of 3 × 10^−7^ M IBTX (IBTX; repeated measures ANOVA: *n* = 7; *P* = .90); (e) effect of 10^−5^ M GoSlo‐SR‐5‐6 on MX‐induced contraction. Vessel tension in the absence (con) and presence of 10^−3^ M TEA (TEA; repeated measures ANOVA: *n* = 5; *P* = .92); (f) effect of 10^−5^ M GoSlo‐SR‐5‐6 on MX‐induced contraction. Vessel tension in the absence (con) and presence of 10^−7^ M penitrem A (PenA; repeated measures ANOVA: *n* = 7; *P* = .91); (g) effect of 3 × 10^−6^ M GoSlo‐SR‐5‐6 on MX‐induced contraction. Vessel tension in the absence (con) and presence of 3 × 10^−7^ M IBTX (IBTX; repeated measures ANOVA: *n* = 8; *P* = .89) and of 10^−7^ M penitrem A (PenA; repeated measures ANOVA: *n* = 8; *P* = .43); (h) effect of 10^−6^ M NS19504 on MX‐induced contraction. Vessel tension in the absence (con) and presence of 10^−7^ M IBTX (IBTX; repeated measures ANOVA: *n* = 8; *P* = .74) and of 10^−7^ M paxillin (Pax; repeated measures ANOVA: *n* = 8; *P* = .82)

Importantly, the relaxant effect of GoSlo‐SR‐5‐6 was abolished after functionally eliminating the influence of K^+^ channels on vessel tension by pre‐constriction with 50 × 10^−3^ M KCl (Figure 6a). Vascular smooth muscle expresses a range of K^+^ channels including BK channels, voltage‐gated potassium channels (K_v_ channels), inward‐rectifying potassium channels and ATP‐sensitive potassium channels (Nelson & Quayle, 1995; Tykocki, Boerman, & Jackson, 2017). As Figure 6b shows, the response to GoSlo‐SR‐5‐6 was abolished after blocking BK channels with their inhibitor https://www.guidetopharmacology.org/GRAC/LigandDisplayForward?ligandId=4218 (IBTX, 10^−7^ M), in combination with the K_v_2 channel blocker https://www.guidetopharmacology.org/GRAC/LigandDisplayForward?ligandId=2576 (STX, 10^−7^ M, Escoubas, Diochot, Celerier, Nakajima, & Lazdunski, 2002), the K_v_1 channel inhibitor, DPO‐1 (10^−6^ M, Lagrutta, Wang, Fermini, & Salata, 2006; Tsvetkov et al., 2016), and the K_v_7 channel blocker XE991 (3 × 10^−6^ M). These data are consistent with the idea that GoSlo‐SR‐5‐6 mediated its effect via activation of K^+^ channels, rather than inhibiting L‐type calcium channels.

The relaxations induced by GoSlo‐SR‐5‐6 on isometric tension recordings were also resistant to BK channel blockade with IBTX. It is clear from the tension record shown in Figure 6c that BK channels were functional in these preparations, since IBTX (3 × 10^−7^ M) constricted these tissues. The response to GoSlo‐SR‐5‐6 (10^−5^ M) was not blocked by the selective BK channel blocker IBTX (Figure 6d,g) and penitrem A (10^−7^ M, Figure 6f,g; Knaus et al., 1994) or low concentrations of the non‐selective K^+^ channel blocker TEA (1 × 10^−3^ M, Figure 6e; Nelson & Quayle, 1995). A similar observation was made for another BK channel opener, NS19504 (Nausch et al., 2014), which induced relaxation not affected by IBTX (10^−7^ M) and paxilline (10^−7^ M; Figure 6h).

**Figure 7:**
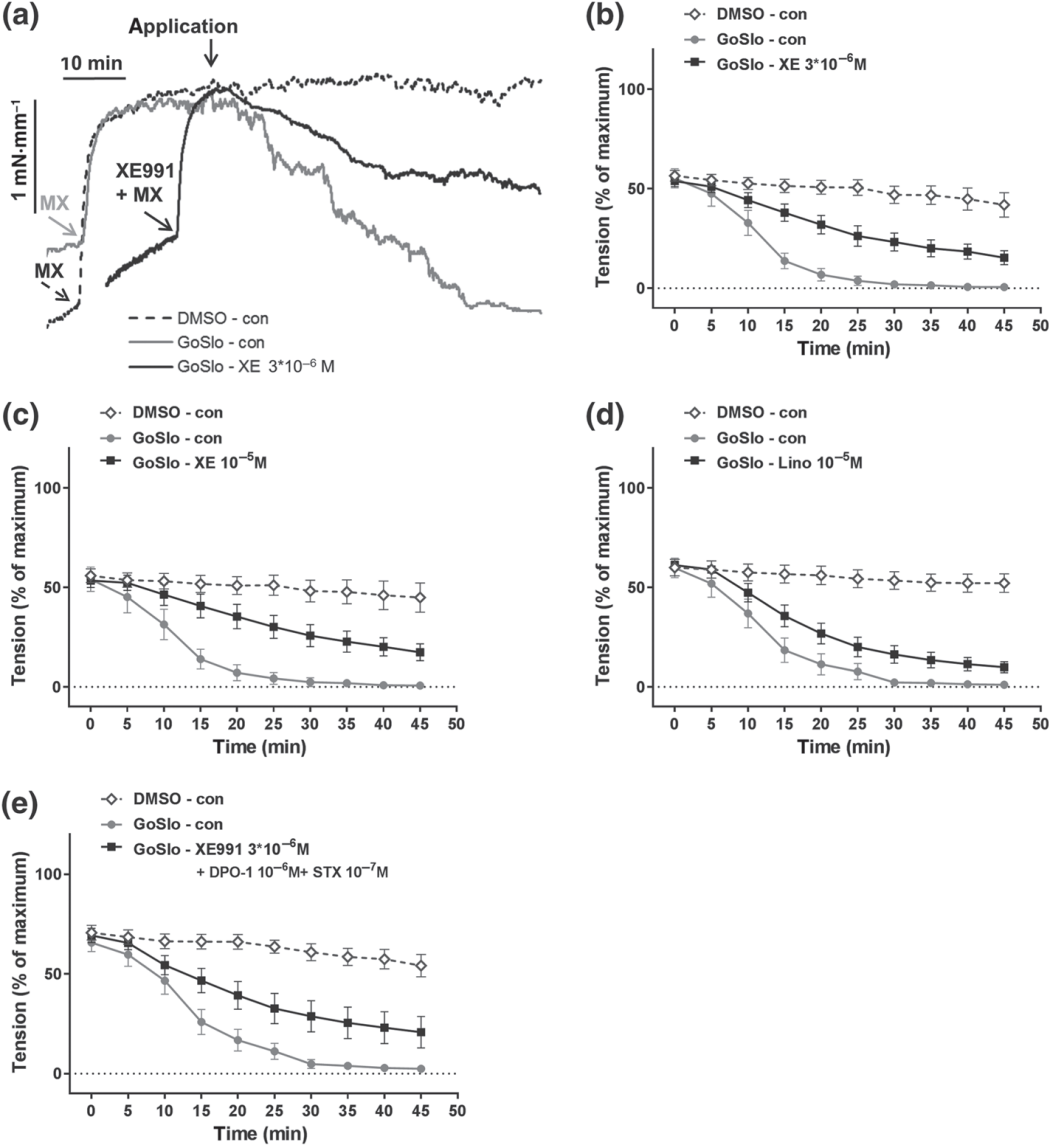
GoSlo‐SR‐5‐6‐induced relaxations of isometric tone are reduced when K_v_7 channels are blocked. (a) Example of the effect of XE991 on relaxation induced by 10^−5^ M GoSlo‐SR‐5‐6. Application denotes the time point where GoSlo‐SR or vehicle was added; (b) effect of 10^−5^ M GoSlo‐SR‐5‐6 on methoxamine (MX)‐induced contraction. Vessel tension in the absence (DMSO–con) and presence of GoSlo‐SR‐5‐6 (GoSlo–con) and of GoSlo‐SR‐5‐6 together with 3 × 10^−6^ M XE991 (GoSlo–XE991; repeated measures ANOVA: GoSlo‐SR‐5‐6 vs. GoSlo‐SR‐5‐6 + XE991: *n* = 9; *P* < .05); (c) effect of 10^−5^ M GoSlo‐SR‐5‐6 on MX‐induced contraction. Vessel tension in the absence (DMSO–con) and presence of GoSlo‐SR‐5‐6 (GoSlo–con) and of GoSlo‐SR‐5‐6 together with 10^−5^ M XE991 (GoSlo–XE991; repeated measures ANOVA: GoSlo‐SR‐5‐6 vs. GoSlo‐SR‐5‐6 + XE991: n = 7; *P* < .05; repeated measures ANOVA: GoSlo‐SR‐5‐6 + 3 × 10^−6^ M XE991 vs. GoSlo‐SR‐5‐6 + 10^−5^ M XE991: *P* = .72); (d) effect of 10^−5^ M GoSlo‐SR‐5‐6 on MX‐induced contraction. Vessel tension in the absence (DMSO–con) and presence of GoSlo‐SR‐5‐6 (GoSlo–con) and of GoSlo‐SR‐5‐6 together with 10^−5^ M linopirdine (GoSlo–Lino; repeated measures ANOVA: GoSlo‐SR‐5‐6 vs. GoSlo‐SR‐5‐6 + Lino: *n* = 9; *P* < .05); (e) effect of 10^−5^ M GoSlo‐SR‐5‐6 on MX‐induced contraction. Vessel tension in the absence (DMSO–con) and presence of GoSlo‐SR‐5‐6 (GoSlo–con) and of GoSlo‐SR‐5‐6 together with 3 × 10^−6^ M XE991, 10^−6^ M DPO‐1, and 10^−7^ M stromatoxin (GoSlo–XE991 + DPO‐1 + STX; repeated measures ANOVA: GoSlo‐SR‐5‐6 vs. GoSlo‐SR‐5‐6 + XE991 + DPO‐1 + STX: *n* = 10; *P* < .05; repeated measures ANOVA: GoSlo‐SR‐5‐6 + 3 × 10^−6^ M XE991 vs. GoSlo‐SR‐5‐6 + XE991 + DPO‐1 + STX: *P* = .25)

Inhibition of K_v_7 channels with XE991 (3 × 10^−6^ M) also considerably reduced, but did not abolish the effect of GoSlo‐SR‐5‐6 on isometric tension (Figure 7a,b). Increasing the concentration of XE991 to 10^−5^ M did not further reduce the effects of GoSlo‐SR‐5‐6 (Figure 7c). Blockade of K_v_7 channels with 10^−5^ M https://www.guidetopharmacology.org/GRAC/LigandDisplayForward?ligandId=2599 also reduced the effect of GoSlo‐SR‐5‐6 (Figure 7d). However, in the presence of 3 × 10^−6^ M XE991, the addition of the K_v_1 channel inhibitor, DPO‐1 (10^−6^ M) together with stromatoxin (STX, 10^−7^ M) did not further reduce the effect of GoSlo‐SR‐5‐6, suggesting that neither K_v_1 nor K_v_2 channels contributed to the vasorelaxant effects of GoSlo‐SR‐5‐6 (Figure 7e).

**Figure 8:**
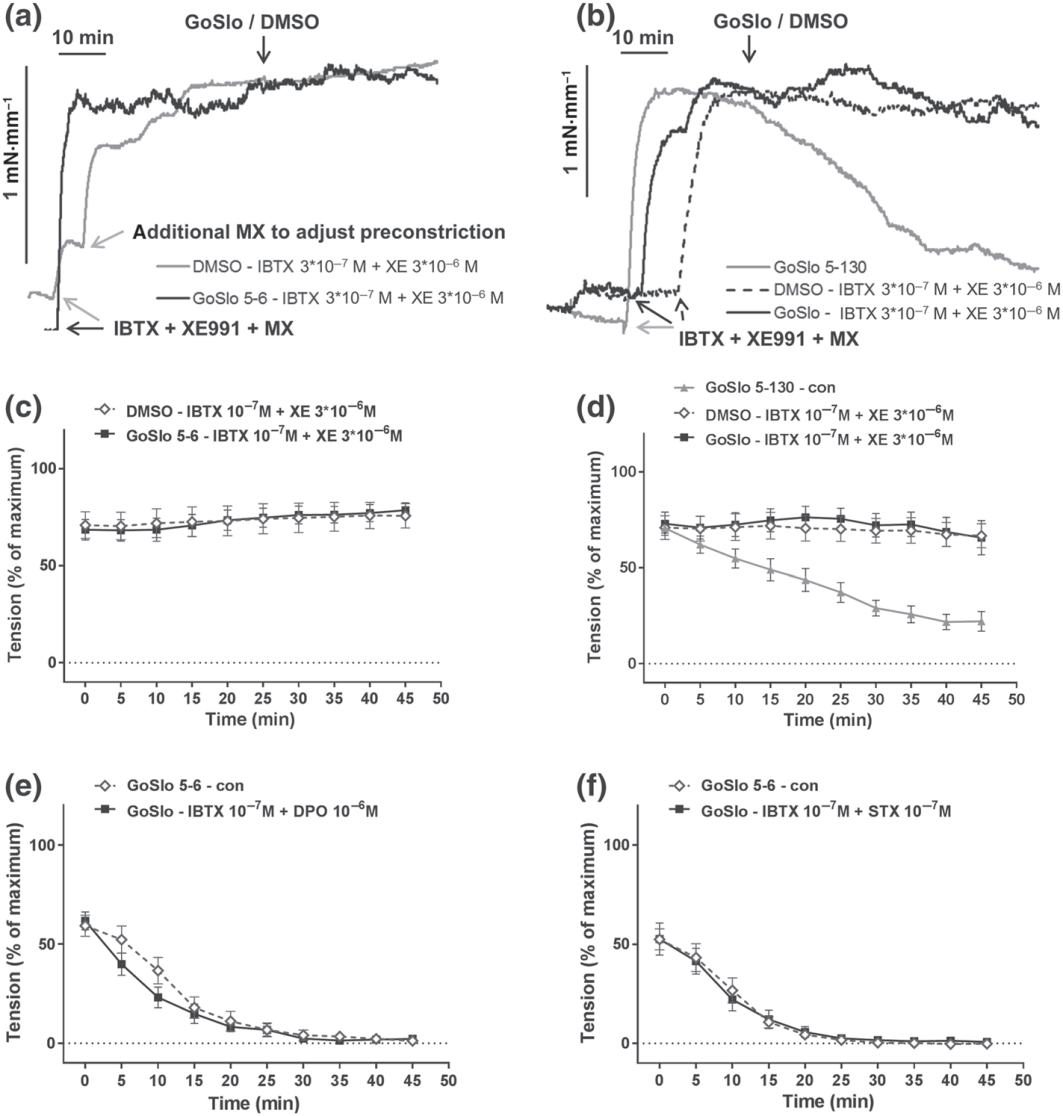
Contribution of BK and K_v_7 channels to the effect of GoSlo‐SR. (a) Example of the effect of IBTX and XE991 on relaxation induced by 10^−5^ M GoSlo‐SR‐5‐6. The arrows with the label (IBTX + XE991 + MX) denote the time points where pre‐constriction was initiated by the addition of IBTX, XE991 and methoxamine (MX). The arrow with the label (GoSlo/DMSO) denotes the time point where GoSlo‐SR‐5‐6 or vehicle (DMSO) was added; (b) example of the effect of IBTX and XE991 on relaxation induced by 10^−5^ M GoSlo‐SR‐5‐130. The arrows with the label (IBTX + XE991 + MX) denote the time points where pre‐constriction was initiated by the addition of IBTX, XE991, and MX. The arrow with the label (GoSlo/DMSO) denotes the time point where GoSlo‐SR‐5‐130 or vehicle (DMSO) was added; (c) effect of 10^−5^ M GoSlo‐SR‐5‐6 on MX‐induced contraction. Vessel tension in the presence of 10^−7^ M IBTX and 3 × 10^−6^ M XE991 in the absence (DMSO) and presence of GoSlo‐SR‐5‐6 (GoSlo; repeated measures ANOVA: *n* = 8; *P* = .98); (d) effect of 10^−5^ M GoSlo‐SR‐5‐130 on MX‐induced contraction. Vessel tension in the presence of 10^−7^ M IBTX and 3 × 10^−6^ M XE991 in the absence (DMSO) and presence of GoSlo‐SR‐5‐130 (GoSlo; repeated measures ANOVA: *n* = 6; *P* = .78); (e) effect of 10^−5^ M GoSlo‐SR‐5‐6 on MX‐induced contraction. Vessel tension in the absence (con) and presence of 10^−7^ M IBTX and 10^−6^ M DPO‐1 (IBTX + DPO; repeated measures ANOVA: *n* = 10; *P* = .43); (f) effect of 10^−5^ M GoSlo‐SR‐5‐6 on MX‐induced contraction. Vessel tension in the absence (con) and presence of 10^−7^ M IBTX and 10^−7^ M stromatoxin (IBTX + STX; repeated measures ANOVA: *n* = 7; *P* = .95)

Of note, as shown in Figure 8, co‐application of IBTX (10^−7^ M) and XE991 (3 × 10^−6^ M) completely abolished the inhibitory effects of GoSlo‐SR compounds on vessels pre‐constricted with MX. Thus, neither GoSlo‐SR‐5‐6 (Figure 8a,c) nor GoSlo‐SR‐5‐130 (Figure 8b,d), applied at a concentration of 10^−5^ M, was able to reduce isometric tension in these experiments, consistent with the idea that GoSlo‐SR compounds mediated their effects by activating both BK and K_v_7 channels.

### 3.3 Effect of GoSlo‐SR compounds on mesenteric, saphenous and tail arteries

**Figure 9:**
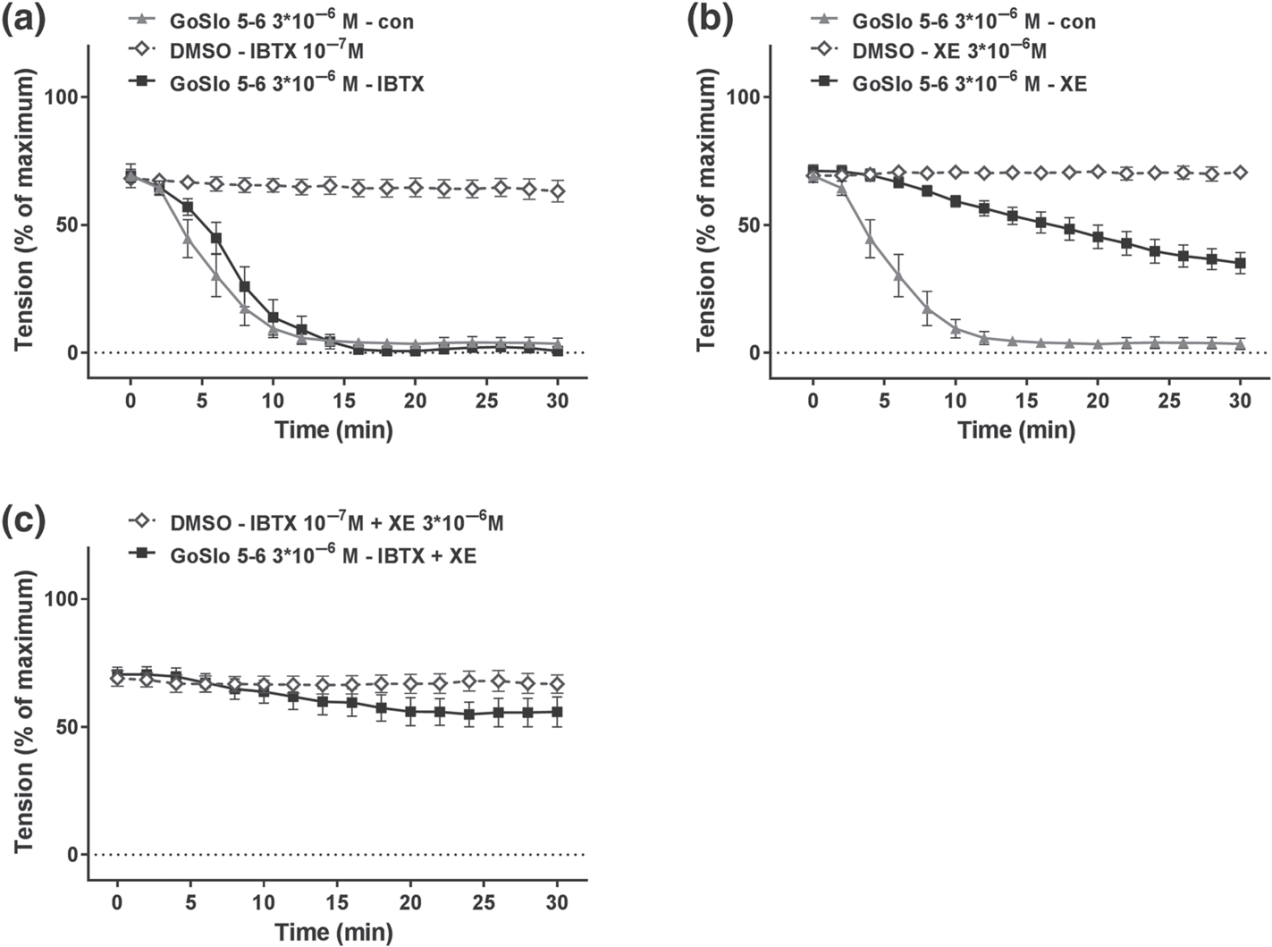
GoSlo‐SR‐5‐6 also causes relaxation of mesenteric arteries. (a) Effect of 3 × 10^−6^ M GoSlo‐SR‐5‐6 on MX‐induced contraction. Vessel tension in the presence of GoSlo‐SR‐5‐6 (GoSlo–con), in the presence of GoSlo‐SR‐5‐6 and 10^−7^ M IBTX (GoSlo–IBTX), and in the presence of the vehicle of GoSlo‐SR and IBTX (DMSO–IBTX). (repeated measures ANOVA: GoSlo‐SR‐5‐6–IBTX vs. DMSO–IBTX: *n* = 7; *P* < .05; GoSlo‐SR‐5‐6–con vs. GoSlo‐SR‐5‐6–IBTX: *n* = 7; *P* = .65); (b) effect of 3 × 10^−6^ M GoSlo‐SR‐5‐6 on MX‐induced contraction. Vessel tension in the presence of GoSlo‐SR‐5‐6 (GoSlo–con), in the presence of GoSlo‐SR‐5‐6 and 3 × 10^−6^ M XE991 (GoSlo–XE), and in the presence of the vehicle of GoSlo‐SR and XE991 (DMSO–XE). (repeated measures ANOVA: GoSlo‐SR‐5‐6–XE vs. DMSO–XE: *n* = 7; *P* < .05; GoSlo‐SR‐5‐6–con vs. GoSlo‐SR‐5‐6–XE: *n* = 7; *P* < .05); (c) effect of 3 × 10^−6^ M GoSlo‐SR‐5‐6 on MX‐induced contraction. Vessel tension in the presence of GoSlo‐SR‐5‐6 and 10^−7^ M IBTX together with 3 × 10^−6^ M XE991 (GoSlo–IBTX + XE) and in the presence of the vehicle of GoSlo‐SR and IBTX together with XE991 (DMSO–IBTX + XE). (repeated measures ANOVA: GoSlo‐SR‐5‐6–IBTX + XE vs. DMSO–IBTX + XE: *n* = 6; *P* = .32)

To understand whether the effect of GoSlo‐SR compounds is unique to the *Gracilis* artery, the effect of GoSlo‐SR‐5‐6 was studied on mesenteric, saphenous, and tail arteries. These arteries have been selected, because they represent different vascular beds and are well studied in the participating laboratories. In mesenteric arteries, 3 × 10^−6^ M GoSlo‐SR‐5‐6 caused relaxation (Figure 9a,b). This response was not blocked by the selective BK channel blocker IBTX (10^−7^ M; Figure 9a), was considerably reduced but not abolished by the K_v_7 channel blocker XE991 (3 × 10^−6^ M; Figure 9b), and was completely abolished by co‐application of IBTX and XE991 (Figure 9c).

**Figure 10:**
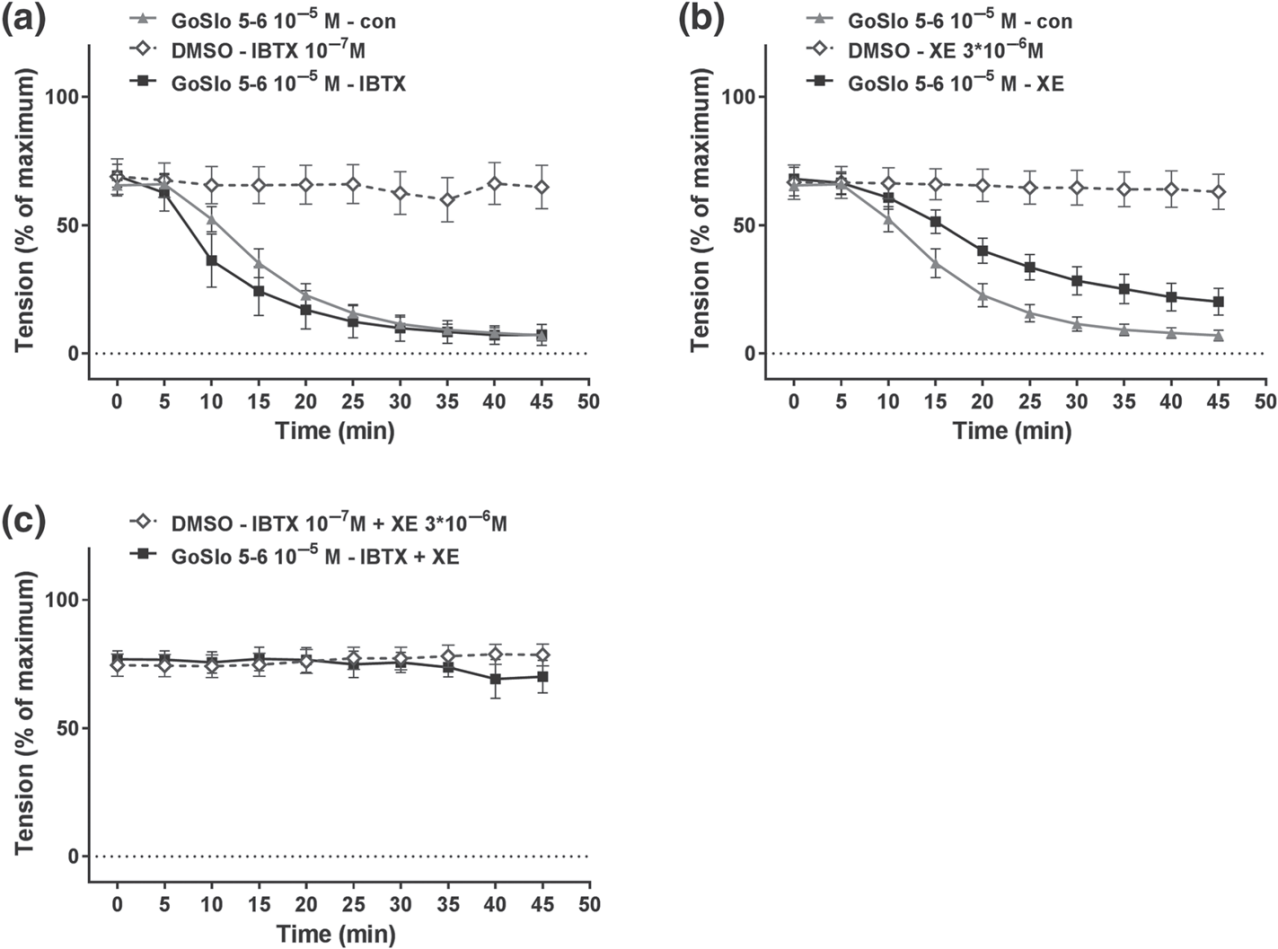
GoSlo‐SR‐5‐6 also causes relaxation of saphenous arteries. (a) Effect of 10^−5^ M GoSlo‐SR‐5‐6 on MX‐induced contraction. Vessel tension in the presence of GoSlo‐SR‐5‐6 (GoSlo–con), in the presence of GoSlo‐SR‐5‐6 and 10^−7^ M IBTX (GoSlo–IBTX), and in the presence of the vehicle of GoSlo‐SR and IBTX (DMSO–IBTX). (repeated measures ANOVA: GoSlo‐SR‐5‐6–IBTX vs. DMSO–IBTX: *n* = 6; *P* < .05; GoSlo‐SR‐5‐6–con vs. GoSlo‐SR‐5‐6–IBTX: *n* = 6; *P* = .55); (b) effect of 10^−5^ M GoSlo‐SR‐5‐6 on MX‐induced contraction. Vessel tension in the presence of GoSlo‐SR‐5‐6 (GoSlo–con), in the presence of GoSlo‐SR‐5‐6 and 3 × 10^−6^ M XE991 (GoSlo–XE), and in the presence of the vehicle of GoSlo‐SR and XE991 (DMSO–XE). (repeated measures ANOVA: GoSlo‐SR‐5‐6–XE vs. DMSO–XE: *n* = 7; *P* < .05; GoSlo‐SR‐5‐6–con vs. GoSlo‐SR‐5‐6–XE: *n* = 7; *P* < .05); (c) effect of 10^−5^ M GoSlo‐SR‐5‐6 on MX‐induced contraction. Vessel tension in the presence of GoSlo‐SR‐5‐6 and 10^−7^ M IBTX together with 3 × 10^−6^ M XE991 (GoSlo–IBTX + XE), and in the presence of the vehicle of GoSlo‐SR and IBTX together with XE991 (DMSO–IBTX + XE). (repeated measures ANOVA: GoSlo‐SR‐5‐6–IBTX + XE vs. DMSO–IBTX + XE: *n* = 7; *P* = .78)

In saphenous arteries, 10^−5^ M GoSlo‐SR‐5‐6 caused relaxation (Figure 10a,b). This response was not blocked by IBTX (10^−7^ M; Figure 10a), was considerably reduced but not abolished by XE991 (3 × 10^−6^ M; Figure 10b), and was completely abolished by co‐application of IBTX and XE991 (Figure 10c).

**Figure 11:**
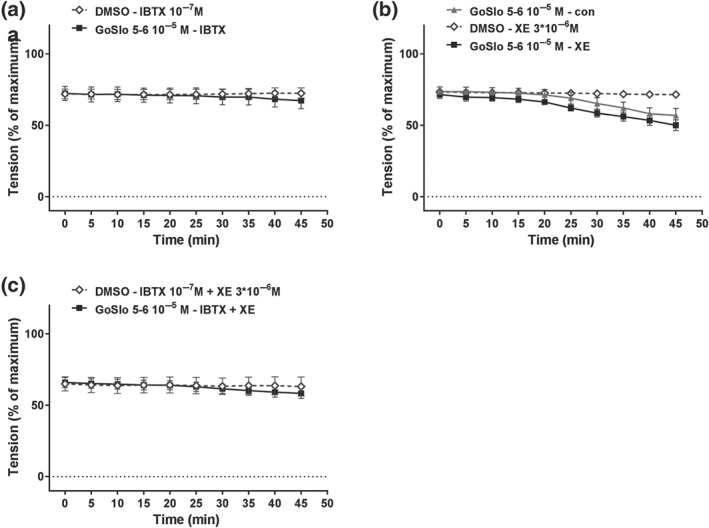
GoSlo‐SR‐5‐6 also causes relaxation of tail arteries. (a) Effect of 10^−5^ M GoSlo‐SR‐5‐6 on MX‐induced contraction. Vessel tension in the presence of GoSlo‐SR‐5‐6 and 10^−7^ M IBTX (GoSlo–IBTX), and in the presence of the vehicle of GoSlo‐SR and IBTX (DMSO–IBTX; for better discrimination of data points vessel tension in the presence of GoSlo‐SR‐5‐6 alone is not shown in this graph—refer to panel (b); repeated measures ANOVA: GoSlo‐SR‐5‐6–IBTX vs. DMSO–IBTX: *n* = 8; *P* = .81); (b) effect of 10^−5^ M GoSlo‐SR‐5‐6 on MX‐induced contraction. Vessel tension in the presence of GoSlo‐SR‐5‐6 (GoSlo–con), in the presence of GoSlo‐SR‐5‐6 and 3 × 10^−6^ M XE991 (GoSlo–XE), and in the presence of the vehicle of GoSlo‐SR and XE991 (DMSO–XE). (repeated measures ANOVA: GoSlo‐SR‐5‐6–XE vs. DMSO–XE: *n* = 7; *P* < .05; GoSlo‐SR‐5‐6–con vs. GoSlo‐SR‐5‐6–XE: *n* = 7; *P* = .23); (c) effect of 10^−5^ M GoSlo‐SR‐5‐6 on MX‐induced contraction. Vessel tension in the presence of GoSlo‐SR‐5‐6 and 10^−7^ M IBTX together with 3 × 10^−6^ M XE991 (GoSlo–IBTX + XE), and in the presence of the vehicle of GoSlo‐SR and IBTX together with XE991 (DMSO–IBTX + XE). (repeated measures ANOVA: GoSlo‐SR‐5‐6–IBTX + XE vs. DMSO–IBTX + XE: *n* = 8; *P* = .85)

In tail arteries, 10^−5^ M GoSlo‐SR‐5‐6 caused relaxation (Figure 11b). This response was completely abolished by IBTX (10^−7^ M; Figure 11a), was not affected by XE991 (3 × 10^−6^ M; Figure 11b), and was completely abolished by co‐application of IBTX and XE991 (Figure 11c).

**Figure 12:**
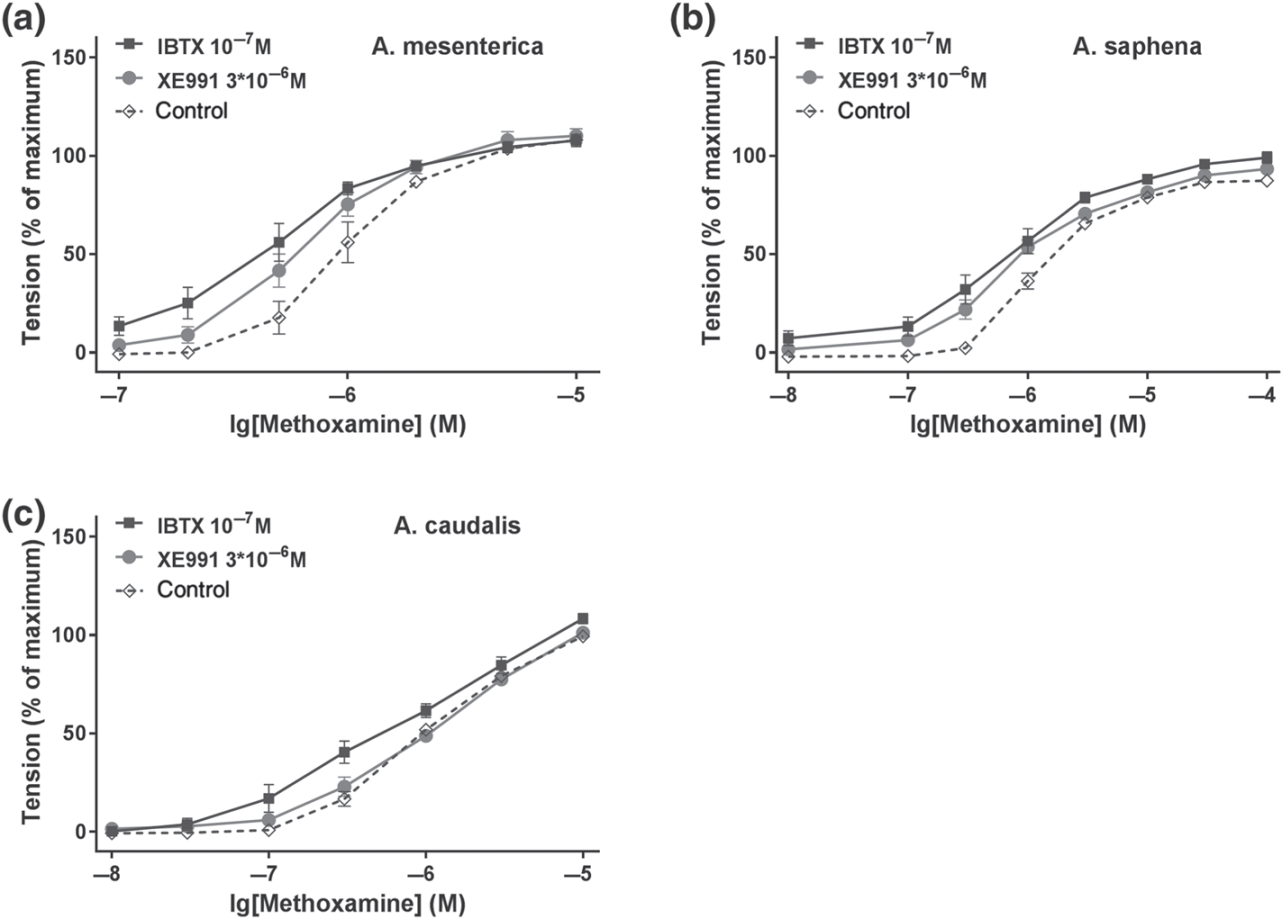
IBTX and XE991 affect methoxamine‐induced contraction. (a) Effect of 10^−7^ M IBTX and 3 × 10^−6^ M XE991 on methoxamine‐induced contraction in mesenteric arteries (repeated measures ANOVA: con vs. IBTX: *n* = 8; *P* < .05; con vs. XE991: *n* = 8; *P* < .05; IBTX vs. XE: *n* = 8; *P* = .20); (b) effect of 10^−7^ M IBTX and 3 × 10^−6^ M XE991 on methoxamine‐induced contraction in saphenous arteries (repeated measures ANOVA: con vs. IBTX: *n* = 9; *P* < .05; con vs. XE991: *n* = 9; *P* < .05; IBTX vs. XE: *n* = 9; *P* = .10); (c) effect of 10^−7^ M IBTX and 3 × 10^−6^ M XE991 on methoxamine‐induced contraction in tail arteries (repeated measures ANOVA: con vs. IBTX: *n* = 7; *P* < .05; con vs. XE991: *n* = 7; *P* = .39)

Of note, the functional availability of BK and K_v_7 channels during contraction induced by MX, the agent used to produce pre‐constriction when the effect of GoSlo‐SR compounds was tested, was observed to be different in these vessels. Thus, in mesenteric (Figure 12a) and saphenous arteries (Figure 12b), both 10^−7^ M IBTX and 3 × 10^−6^ M XE991 increased methoxamine‐induced contractile responses to a similar degree. In contrast, in tail arteries, this effect was observed only for IBTX (Figure 12c).

### 3.4 Effect of GoSlo‐SR compounds on membrane potential and BK currents

**Figure 13:**
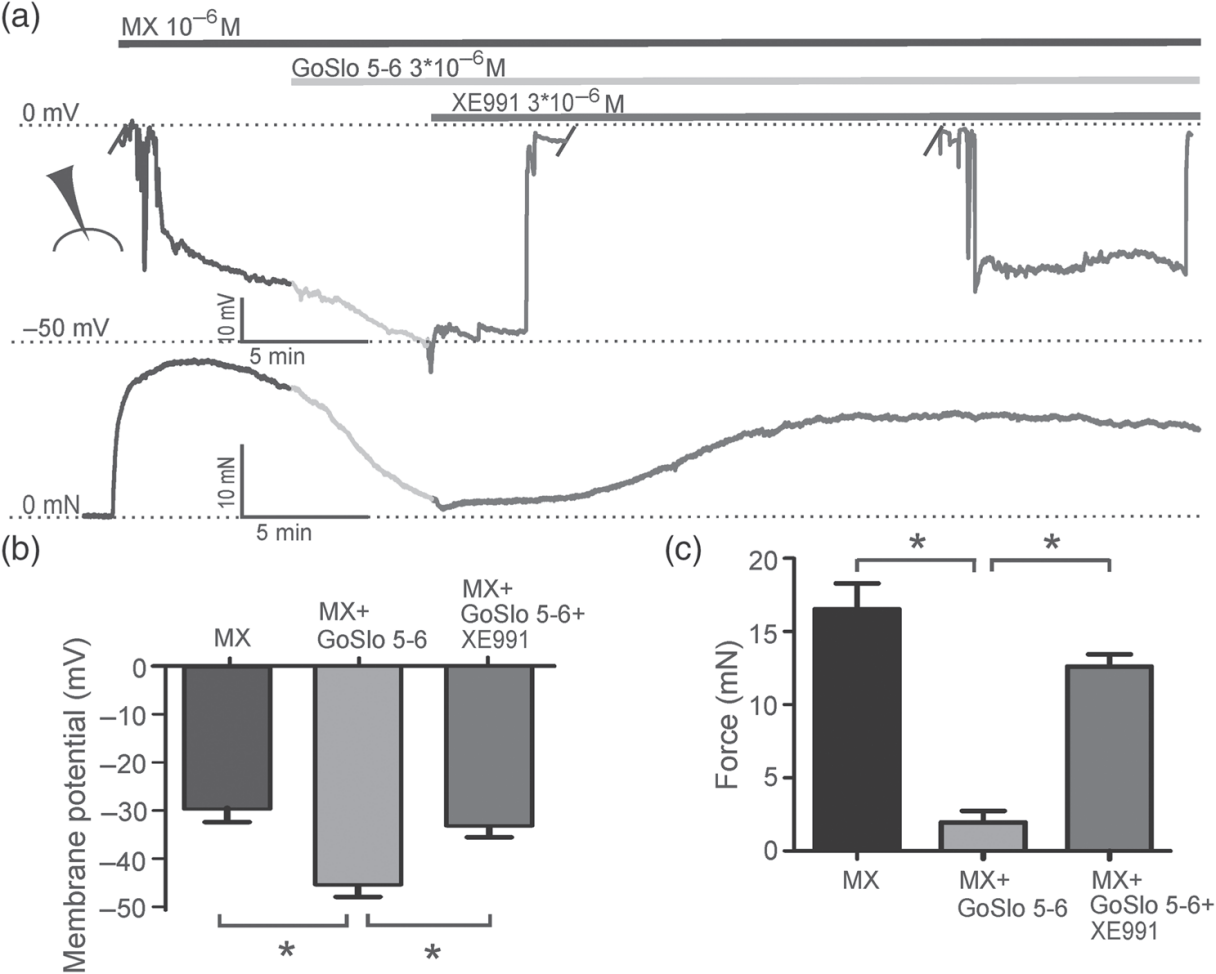
GoSlo‐SR‐5‐6 causes hyperpolarisation of isometric preparations of rat mesenteric arteries. (a) Example of the effect of 3 × 10^−6^ M GoSlo‐SR‐5‐6 on membrane potential (upper trace) and contractile force (lower trace) of an isometric vessel preparation at 10^−6^ M methoxamine (MX)‐induced tone and after subsequent application of 3 × 10^−6^ M XE991. The microelectrode symbol denotes phases when the microelectrode was impaled/not impaled. Summarised data of membrane potential (b) and contractile force (c) in the presence of 10^−6^ M MX, MX + 3 × 10^−6^ M GoSlo‐SR‐5‐6, and MX + GoSlo‐SR‐5‐6 + 3 × 10^−6^ M XE991. (^*^repeated measures ANOVA: MX vs. MX–GoSlo‐SR‐5‐6(*n* = 7); *P* < .05; MX–GoSlo‐SR‐5‐6 vs. MX–GoSlo‐SR‐5‐6–XE(*n* = 6); *P* < .05, Bonferroni's multiple comparison test)

**Figure 14:**
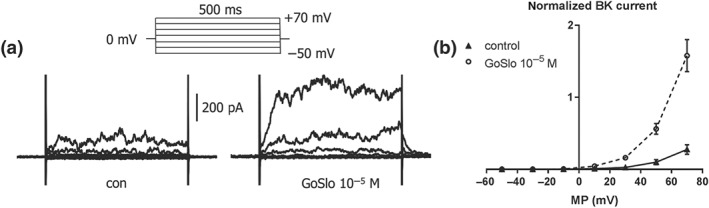
GoSlo‐SR‐5‐6 stimulates BK currents in freshly isolated smooth muscle cells of rat tail artery. (a) Example of the effect of 10^−5^ M GoSlo‐SR‐5‐6 on BK currents. Example traces of the BK current at different voltages in the absence (con, left panel) and presence of GoSlo‐SR‐5‐6 (GoSlo, right panel); the inset shows the voltage protocol—it consisted of 500‐ms‐long voltage steps from a holding potential of 0 mV to test potentials between −50 and +70 mV in 20‐mV increments applied every 5 s. (b) Summarised current–voltage (MP) relationship of the BK current in the absence (Control) and presence of GoSlo‐SR‐5‐6 (GoSlo). (repeated measures ANOVA: control vs. GoSlo‐SR‐5‐6: *n* = 6; *P* < .05)

To get more direct evidence for the involvement of ion channels in the relaxations induced by GoSlo‐SR compounds, electrophysiological experiments were performed. We first measured the membrane potential in smooth muscle cells of intact mesenteric arteries using microelectrodes. The mesenteric artery was selected for these experiments because it was possible to get reliable membrane potential measurements (for criteria, see Section 2.6). We observed that the GoSlo‐SR‐5‐6‐induced relaxation was associated with a hyperpolarisation from −29 ± 4 to −45 ± 3 mV (*n* = 7; Figure 13). We were able to reverse this hyperpolarisation and relaxation with the K_v_7 channel blocker XE991 (Figure 13). In a few of these vessels, we were also able to measure the membrane potential after the subsequent addition of IBTX, in the continued presence of XE991. In these preliminary experiments (*n* = 3), membrane potential was −33 ± 2 mV in XE991 and −27 ± 1 mV after both XE991 and IBTX application, suggesting that BK channels did contribute to the hyperpolarisation. However, these experiments were difficult to perform, and impalements were often lost in XE991 and IBTX, due to the induction of spontaneous activity by these ion channel blockers. To explore the role of BK channels further, we also measured BK currents in freshly isolated tail artery smooth muscle cells, which have been well documented to possess BK currents (Schubert, Noack, & Serebryakov, 1999; Schubert, Serebryakov, Engel, & Hopp, 1996). We observed that GoSlo‐SR‐5‐6 induced a considerable increase in BK currents (Figure 14). A similar increase in BK currents was obtained in freshly isolated rat mesenteric artery smooth muscle cells studied. However, a pronounced current run‐down precluded more detailed studies with these cells.

### 3.5 Effect of GoSlo‐SR compounds on K_v_7.4 and K_v_7.5 channels

**Figure 15:**
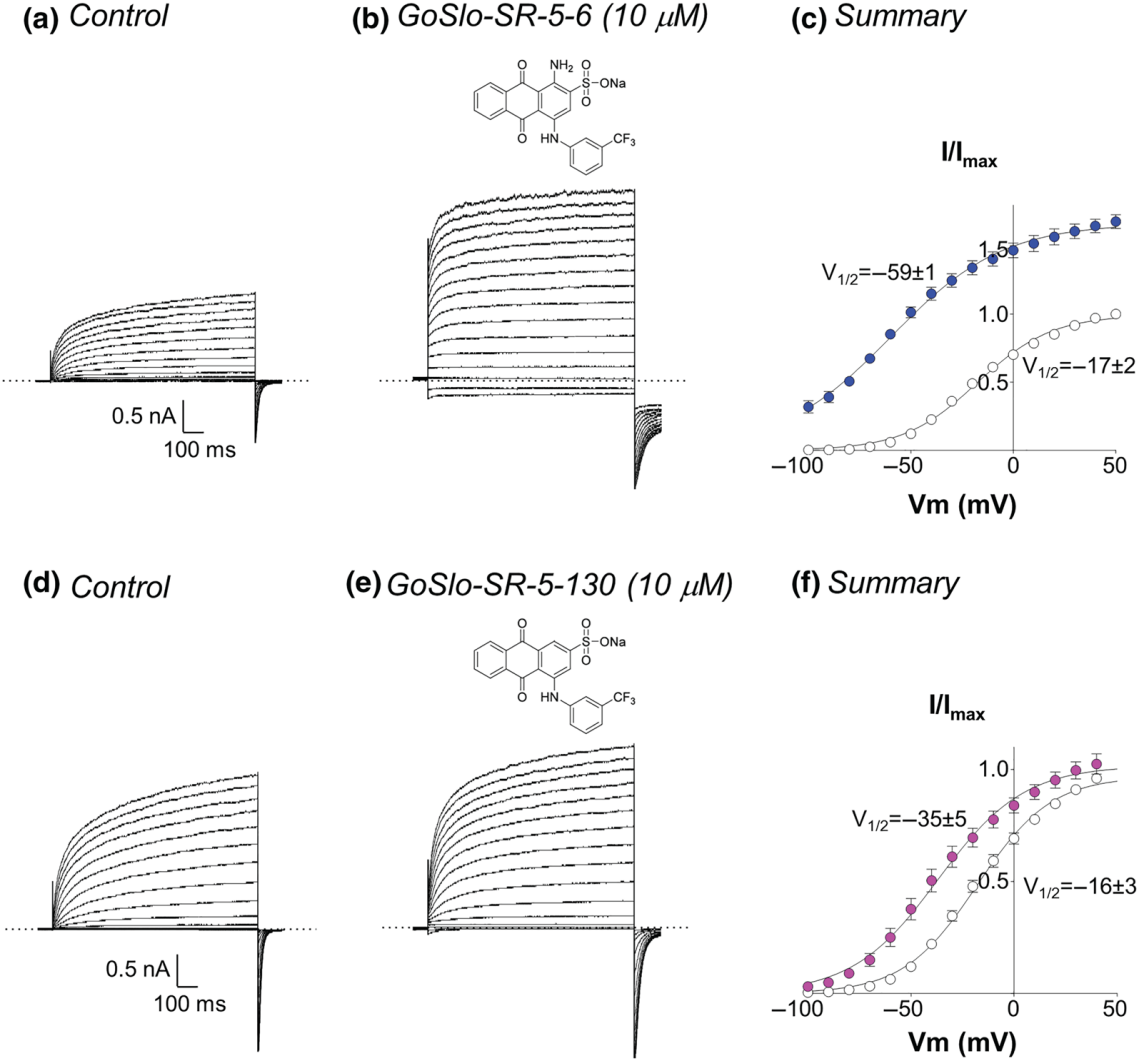
GoSlo‐SR compounds activate K_v_7.4 channels. (a) Typical family of currents obtained from a HEK cell during a series of voltage steps from −100 to +60 mV in 10‐mV increments lasting 1 s. Cells were held at −80 mV and repolarised back to −120 mV to obtain tail currents; (b) currents from the same cell during incubation with 10^−5^ M GoSlo‐SR‐5‐6. Tail current deactivation (τ) recorded at −120 mV following a step to +40 mV increased from 15 ± 1 ms to 47 ± 5 ms (*n* = 6; *P* < .05; paired Student's *t‐*test); (c) summary activation curves obtained by measuring tail currents in six cells before (open circles) and during (blue circles) application of GoSlo‐SR‐5‐6; (d) and (e) currents obtained from a different cell, held at −80 mV and stepped from −100 mV to +50 mV in 10 mV increments, in the absence and presence of GoSlo‐SR‐5‐130 (10^−5^ M), respectively. Tail currents recorded at −120 mV following a step to +40 mV decayed with a τ of 15 ± 2 ms in control conditions compared to 26 ± 1 ms in GoSlo‐SR‐5‐130 (*n* = 6; *P* < .05; paired *t* test); (f) summary activation curves obtained from six cells in the absence (open circles) and presence (pink circles) of GoSlo‐SR‐5‐130

To test if GoSlo‐SR compounds activated K_v_7 channels, we examined the effects of extracellularly applied GoSlo‐SR‐5‐6 (10^−5^ M) on whole cell currents recorded from HEK cells transiently transfected with human K_v_7.4 cDNA. Preliminary experiments established that the EC_50_ for GoSlo‐SR‐5‐6 on K_v_7.4 applied at a potential of −40 mV was 6.4 × 10^−6^ ± 0.5 μM (*n* = 5), suggesting that it was slightly less potent on K_v_7.4 channels, compared to BK channels (2.3 × 10^−6^ μM) reported previously (Roy et al., 2012). Figure 15a shows a family of currents recorded from a cell expressing K_v_7.4 channels. In the absence of any drugs, these currents activated slowly at potentials positive to −60 mV (Figure 15c). Application of GoSlo‐SR‐5‐6 (10^−5^ M) increased current amplitude at all voltages tested and dramatically slowed tail current deactivation (Figure 15b). These effects were reversible on washout, and the currents were blocked in the presence of XE991 (10^−5^ M, exploratory data, *n* = 4, data not shown). The activation curve in the presence of GoSlo‐SR‐5‐6 (10^−5^ M) was characterised by a shift of V_1/2_ by approximately −40 mV (*n* = 6; *P* < .05; paired *t* test); the slope factor was increased from 18 ± 1 mV to 28 ± 2 mV (*n* = 6; *P* < .05; paired *t* test; Figure 15c). It is important to note that the application of GoSlo‐SR‐5‐6 increased the amplitude of the peak tail current at all potentials recorded. It is clear from these data that GoSlo‐SR‐5‐6 activates K_v_7.4 channels and shifts their voltage‐dependent activation to more negative potentials. Another K_v_7 channel activator, ML213 (10^−5^ M), also significantly shifted the activation *V*_1/2_ by −35 ± 2 mV (from −20 ± 2 mV to −54 ± 2 mV, ) and increased *G*_max_ to 2.2 ± 0.2 and these effects were abolished in the W242L mutant (*n* = 5, data not shown). In contrast, the effects of GoSlo‐SR‐5‐6 were not altered in K_v_7.4 channels with a W242L mutant (*n* = 5, data not shown), suggesting that, in contrast to https://www.guidetopharmacology.org/GRAC/LigandDisplayForward?ligandId=2601, residue W242 was not essential for GoSlo‐SR compounds to mediate their effects.

GoSlo‐SR‐5‐130 (10^−5^ M) also activated K_v_7.4 channels (Figure 15,e,f), but it was clearly less efficacious than GoSlo‐SR‐5‐6 (Figure 15a,b,c). We were unable to determine an EC_50_ for this compound on K_v_7.4 because maximal effects were not reached at 30 μM and the compound came out of solution at higher concentrations. Nevertheless, we only observed a small increase in the steady state current amplitude with 10^−5^ M GoSlo‐SR‐5‐130. However, the tail currents were clearly slowed compared to the control currents. GoSlo‐SR‐5‐130 significantly shifted the activation *V*_1/2_ by approximately −20 mV (*n* = 6) , and this was significantly less effective than GoSlo‐SR‐5‐6 (Δ*V*_1/2_ approximately −40 mV). The slope factor K was unaffected (19 ± 1 mV under control conditions and 21 ± 2 mV in GoSlo‐SR‐5‐130).

**Figure 16:**
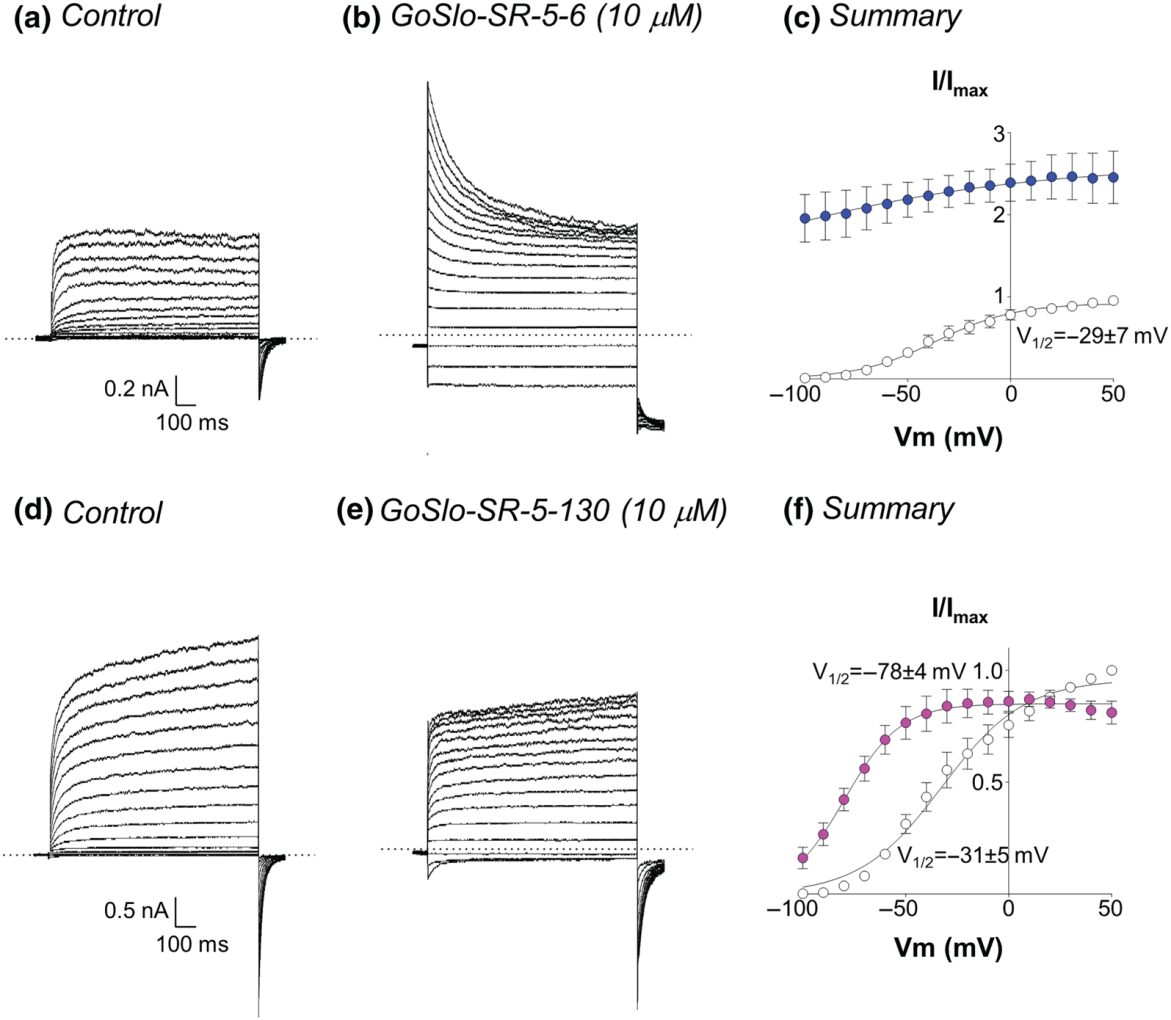
GoSlo‐SR compounds activate K_v_7.5 channels. (a) Typical family of currents obtained from a HEK cell during a series of voltage steps from −100 to +50 mV in 10‐mV increments lasting 1 s. Dotted lines represent the zero current level. Cells were held at −80 mV and repolarised back to −120 mV to obtain tail currents; (b) currents from the same cell during incubation with 10^−5^ M GoSlo‐SR‐5‐6; (c) summary activation curves obtained by measuring tail currents in six cells before (open circles) and during (blue circles) application of GoSlo‐SR‐5‐6; (d) and (e) currents obtained from a different cell, held at −80 mV and stepped from −100 to +50 mV in 10‐mV increments, in the absence and presence of GoSlo‐SR‐5‐130 (10^−5^ M), respectively. Tail current deactivation increased from 26 ± 4 ms to 46 ± 4 ms following a repolarisation from +40 to −120 mV (*n* = 5; *P* < .05; paired *t* test); (f) summary activation curves obtained from five cells in the absence (open circles) and presence (pink circles) of GoSlo‐SR‐5‐130

We next examined the effects of the two GoSlo‐SR compounds on HEK cells expressing K_v_7.5 channels. We were unable to determine the EC_50_ of either GoSlo‐SR‐5‐6 or 5‐130 on K_v_7.5 due to limited solubility of these compounds in Hank's solution at concentrations above 30 μM. A brief inspection of the current amplitude in the first 50 ms demonstrates that GoSlo‐SR‐5‐6 (10^−5^ M) increased the amplitude of the K_v_7.5 current at all voltages, but this effect was particularly apparent at negative potentials (Figure 16a,b). At potentials positive to −60 mV, it is clear that although GoSlo‐SR‐5‐6 increased the initial current amplitude, the currents decreased during the depolarising pulse, presumably as a result of open channel block. When the cell was repolarised from positive potentials back to −120 mV, the apparent block was relieved, and the tail current amplitude consequently increased over time. The activation curve of the control current was constructed from tail currents measured 100 ms after the repolarisation step began, to minimise any distortion of the relationship caused by the block at positive potentials and was characterised by a *V*_1/2_ of −29 ± 7 mV and the slope factor was 19 ± 4 mV. It was not possible to measure either *V*_1/2_ or slope factor in the presence of GoSlo‐SR‐5‐6, as the activation curve was approximately linear over the entire voltage range recorded. What is clear, however, is that the current amplitude in GoSlo‐SR‐5‐6 was much greater at every voltage recorded, compared to control.

GoSlo‐SR‐5‐130 also activated K_v_7.5 currents (Figure 16d–f), but it was less efficacious than GoSlo‐SR‐5‐6 (Figure 16a–c) as evidenced by their effects on *G*_max_. Thus, in GoSlo‐SR‐5‐130, *G*_max_ was 0.9 ± 0.03, and this was significantly less than that recorded in GoSlo‐SR‐5‐6 (2.9 ± 0.2, *n* = 5). Although GoSlo‐SR‐5‐130 (10^−5^ M) slowed the tail currents and increased the tail currents evoked following steps to more negative potentials (<0 mV), it also slightly reduced their peak amplitude following depolarisations positive to +10 mV (Figure 16e). Further, it significantly shifted *V*_1/2_ by approximately −50 mV (slope factors were unaffected 20 ± 3 mV in control compared to 18 ± 4 mV in the presence of the drug (Figure 16f)).

## 4 DISCUSSION

We utilised isometric tension and isobaric diameter recordings of rat arteries to examine the effects of two recently disclosed BK channel openers on vascular smooth muscle. Our focus was to ascertain if these compounds relaxed vascular smooth muscle and determine if these effects were mediated exclusively through activation of BK channels.

### 4.1 Effect of GoSlo‐SR compounds on rat arteries

Two different GoSlo‐SR compounds, GoSlo‐SR‐5‐6 and GoSlo‐SR‐5‐130, produced a strong relaxation of isobaric as well as isometric preparations of rat *Gracilis* muscle arteries. To the best of our knowledge, the effects of GoSlo‐SR compounds on blood vessels have not been reported before. These findings are supported by the reports demonstrating that GoSlo‐SR‐5‐130 reduced spontaneous contractility in rabbit visceral smooth muscle. However, both GoSlo‐SR compounds did not affect induced contractility in these preparations (Hannigan et al., 2016; Large et al., 2015). Taken together, these findings show that GoSlo‐SR compounds are much more effective relaxants in vascular compared to visceral smooth muscle.

Previous studies showed that some effects of GoSlo‐SR compounds on visceral smooth muscle contractility are abolished by pretreatment with specific BK channel blockers (Hannigan et al., 2016; Large et al., 2015). Consequently, in the present study on vascular smooth muscle, we tested whether K^+^ channels in general mediate the effect of GoSlo‐SR compounds. The influence of K^+^ channels on vessel tension was functionally eliminated by pre‐constricting the vessels with 50 mM KCl. At this extracellular KCl concentration, the equilibrium potential for K^+^ is close to the actual membrane potential of smooth muscle cells. Thus, the driving force for potassium ions is negligible. Even if K^+^ channels were open, there would be no K^+^ efflux, hence no alteration of the membrane potential and no change in contractility. Importantly, vasodilators acting on other mechanisms except K^+^ channels would retain their ability to affect vessel tone; only K^+^ channel openers would lose this capability. Indeed, this was observed; the GoSlo‐SR compounds were without any effect after pre‐constriction of the vessels with 50 mM KCl. Moreover, the same effect was seen after blocking functionally important K^+^ channels in vascular smooth muscle (Nelson & Quayle, 1995; Tykocki, Boerman, & Jackson, 2017) using the BK channel inhibitor iberiotoxin (Galvez et al., 1990), the K_v_2 channel blocker stromatoxin (Escoubas, Diochot, Celerier, Nakajima, & Lazdunski, 2002), the K_v_1 channel inhibitor DPO‐1 (Lagrutta, Wang, Fermini, & Salata, 2006; Tsvetkov et al., 2016) and the K_v_7 channel blocker XE991 (Greenwood & Ohya, 2009). The fact that the GoSlo‐SR compounds were unable to relax blood vessels after either treatment supports the idea that GoSlo‐SR compound‐induced vasodilation was due to activation of K^+^ channels. This is further supported by previous findings showing that GoSlo‐SR‐5‐6 and 5‐130 had no significant effect on smooth muscle L‐type calcium currents (Large et al., 2015), that GoSlo‐SR‐5‐130 was not able to affect contractile activity in rabbit bladder in the presence of IBTX (Large et al., 2015) and that GoSlo‐SR‐5‐130 had no effect on KCl‐induced contractions in rabbit corpus cavernosum (Hannigan et al., 2016).

In conclusion, the data presented in this study show that GoSlo‐SR compounds mediate their vasodilator effects exclusively by activation of K^+^ channels, without the involvement of other vasodilator pathways, for example, voltage‐gated calcium channels.

### 4.2 Role of BK channels in mediating the effect of GoSlo‐SR compounds on rat arteries

Recent publications (Hannigan et al., 2016; Large et al., 2015) suggest that the inhibitory effects of the GoSlo‐SR family of compounds on urogenital smooth muscles are mediated by activation of BK channels. Thus, we hypothesised that BK channels may play a leading role in the effect of GoSlo‐SR compounds on vascular smooth muscle. Unexpectedly, we were, initially, unable to get any direct support for an involvement of BK channels in the effect of GoSlo‐SR compounds on vascular smooth muscle, despite evidence that BK channels were functional in these preparations. Thus, pretreatment of *Gracilis*, mesenteric, or saphenous arteries with the most specific BK channel inhibitor, iberiotoxin (Galvez et al., 1990) did not alter the vasodilation induced by the GoSlo‐SR compounds, even when IBTX was applied at a high concentration (3 × 10^−7^ M). However, it is important to note that IBTX alone abolished the GoSlo‐SR‐induced relaxation in rat tail artery, which, as discussed later, is because K_v_7 channels are functionally unavailable in these vessels.

In the *Gracilis* artery, two other widely used BK channel inhibitors, TEA at 10^−3^ M, a concentration affecting primarily BK channels (Nelson & Quayle, 1995), and the specific BK channel inhibitor penitrem A at 10^−7^ M (Knaus et al., 1994), were also unable to modify the effect of the GoSlo‐SR compounds. Finally, we considered the possibility that the BK channel blocker may affect the efficiency of the GoSlo‐SR compounds to dilate vessels but not their maximal effect at saturating concentrations. However, neither IBTX nor penitrem A altered the effect of the GoSlo‐SR compound at a lower concentration not producing full vasodilation. These data suggested that GoSlo‐SR compounds either interfered with the binding of BK channel blockers or that they activated other K^+^ channels. The former explanation appears unlikely, given that the effects of GoSlo‐SR compounds have been shown to be blocked with IBTX and penitrem A in tissue strips (Hannigan et al., 2016; Large et al., 2015) and in single cells (Webb et al., 2015) and that in our study IBTX inhibited the effect of GoSlo‐SR compounds in the tail artery and regained a blocking effect against GoSlo‐SR compounds in the presence of the K_v_7 channel inhibitor XE991 (for more details, see below).

In conclusion, the data presented in this study show that GoSlo‐SR compounds mediated their vasodilator effects exclusively by activating K^+^ channels but are not consistent with the idea that activation of BK channels is the predominant mechanism mediating their effect in either rat *Gracilis*, mesenteric or saphenous arteries.

### 4.3 Role of K_v_7 channels in the effect of GoSlo‐SR compounds on rat arteries

In view of our conclusion that other K^+^ channels, in addition to BK channels, mediate the vasodilator effect of GoSlo‐SR compounds, we hypothesised that K_v_7 channels may play a leading role in this effect. This idea was based on the fact that K_v_7 channels are well‐known to be expressed in a large variety of vascular smooth muscle and to be involved in vasoconstriction and vasodilation (see recent reviews of Barrese, Stott, & Greenwood, 2018; Byron & Brueggemann, 2018; Haick & Byron, 2016; Tykocki, Boerman, & Jackson, 2017). In particular, transcriptional expression of K_v_7 channel genes and their involvement in the regulation of vessel contractility has been shown for rat mesenteric (Jepps et al., 2011; Jepps, Carr, Lundegaard, Olesen, & Greenwood, 2015; Yeung et al., 2007), *Gracilis* muscle (Zavaritskaya et al., 2013), and saphenous (Shvetsova, Gaynullina, Tarasova, & Schubert, 2019) arteries. We confirmed the previous findings regarding the transcriptional expression of K_v_7 genes using an alternative approach, digital PCR, reproducing our previous data that in rat *Gracilis* muscle arteries, like in many other arteries (see recent reviews of Barrese, Stott, & Greenwood, 2018; Byron & Brueggemann, 2018; Haick & Byron, 2016; Tykocki, Boerman, & Jackson, 2017), both KCNQ4 and KCNQ5 channel genes showed higher levels of expression than the other KCNQs.

Furthermore, we obtained novel data proposing an involvement of K_v_7 channels in the effect of GoSlo‐SR compounds on vascular smooth muscle. Thus, the widely used K_v_7 channel inhibitor XE991 (Greenwood & Ohya, 2009; Zavaritskaya et al., 2013) reduced the vasodilating, as well as the hyperpolarising effects of the GoSlo‐SR compounds considerably. XE991 has been employed with great success to identify specific roles of KCNQ‐encoded channels in the circulatory system (Greenwood & Ohya, 2009; Mackie & Byron, 2008). Of note, we observed that the partial inhibition of the GoSlo‐SR‐induced dilation by XE991 was not altered further after elevating the concentration of XE991 from 3 × 10^−6^ to 10^−5^ M, suggesting that activation of K_v_7 channels was not the only mechanism mediating GoSlo‐SR‐induced vasodilation.

The GoSlo‐SR‐induced vasodilation that remained in the presence of XE991 was not affected when XE991 was co‐applied with either the specific K_v_1 channel inhibitor DPO‐1 (Lagrutta, Wang, Fermini, & Salata, 2006; Tsvetkov et al., 2016) or the specific K_v_2 channel inhibitor stromatoxin (Escoubas, Diochot, Celerier, Nakajima, & Lazdunski, 2002). K_v_1 and K_v_2 channels are the other major K_v_ channel subtypes expressed in arterial smooth muscle (Albarwani et al., 2003; Amberg & Santana, 2006). Thus, either K_v_1 or K_v_2 channels are not activated by the GoSlo‐SR compounds or these K_v_ channels are not functionally available in rat *Gracilis* arteries. The latter explanation seems unlikely, because we have observed in an ongoing study that DPO‐1 and stromatoxin are able to modify myogenic constriction of this vessel (data not published). Thus, K_v_1 and K_v_2 channels are functionally available in rat *Gracilis* arteries but are not involved in the vasorelaxant effects of GoSlo‐SR compounds.

Based on the transcriptional expression data, our results suggest that the GoSlo‐SR‐induced vasodilation in *Gracilis* muscle, mesenteric and saphenous arteries is mediated mainly by K_v_7.4 and K_v_7.5 channels. We excluded any contribution from K_v_7.1 channels in this response since the specific K_v_7.1 channel inhibitor https://www.guidetopharmacology.org/GRAC/LigandDisplayForward?ligandId=2590 (Chadha et al., 2012; Gogelein, Bruggemann, Gerlach, Brendel, & Busch, 2000) failed to affect the vasodilatory activity of GoSlo‐SR‐5‐6.

Importantly, we observed that GoSlo‐SR compounds activated K_v_7.4 and K_v_7.5 currents, an effect associated with a shift of the activation properties of these channels to more negative potentials. In this respect, GoSlo‐SR‐5‐130, the GoSlo‐SR compound with weaker vasodilating capacity, appeared to be much less efficacious than GoSlo‐SR‐5‐6. Taken together, our data strongly suggest that activation of K_v_7.4 and/or K_v_7.5 channels or of K_v_7.4/7.5 heteromeric channels (Brueggemann et al., 2014; Chadha et al., 2014) contribute to the vasorelaxant effects of GoSlo‐SR compounds.

### 4.4 Role of BK and K_v_7 channels in the effect of GoSlo‐SR compounds on rat arteries

As discussed so far, blockade of K_v_7 channels only partially reduced the effect of the GoSlo‐SR compounds in *Gracilis*, mesenteric and saphenous arteries. However, the combined application of IBTX and XE991 to either isobaric or isometric vessel preparations abolished the vasodilating effect of the GoSlo‐SR compounds completely. This supports the idea that they relaxed *Gracilis* muscle arteries by activating both BK and K_v_7 channels. This dual action of GoSlo‐SR compounds on BK and K_v_7.4/K_v_7.5 channels is further supported by the findings made on single cells expressing these channels, that is, (a) our findings reported in the present study demonstrating that GoSlo‐SR compounds produce a large stimulation of native BK currents and K_v_7.4 and K_v_7.5 channels and (b) previously published findings showing that GoSlo‐SR compounds activate expressed as well as native BK channels (Hannigan et al., 2016; Kshatri et al., 2017; Large et al., 2015; Roy et al., 2012; Roy et al., 2014; Webb et al., 2015). Incidentally, the BK channel opener NS11021 has been shown to stimulate expressed K_v_7.4 channels (Bentzen et al., 2007), and BMS204352 activates both BK and K_v_7 channels (Schroder, Strobaek, Olesen, & Christophersen, 2003). Thus, the joint activation of BK and K_v_7.4/K_v_7.5 channels by BK channel opener compounds is not without precedent and perhaps suggests that they interact with a common site on both BK and K_v_ channels. Future studies will be focused at determining the precise location of this site in K_v_7 channels.

Importantly, the degree of contribution of BK and K_v_7.4/K_v_7.5 channels to the GoSlo‐SR compound‐induced vasodilation varied depending on the experimental conditions. Thus, GoSlo‐SR compound‐induced vasodilation (a) was not affected by inhibition of BK channels when K_v_7.4/7.5 channels were not blocked (b) but was completely abolished by inhibition of BK channels when K_v_7.4/7.5 channels were blocked. Of note, the latter finding is emphasised by our data on tail arteries. In this artery, in contrast to all other vessels studied, K_v_7 channels appeared to be functionally unavailable during MX‐induced contraction, as evidenced by the absence of an effect of XE991 on this contraction (see Figure 12c). Here, again in contrast to the *Gracilis*, mesenteric, and saphenous arteries, the GoSlo‐SR compound‐induced vasodilation was completely abolished when BK channels alone were blocked. Together, this suggests that when K_v_7.4/7.5 channels are not functionally available, the effect of GoSlo‐SR compounds on BK channels was sufficient to relax the blood vessels. However, when K_v_7.4/7.5 channels were functionally available, blockade of BK channels failed to reduce the response to GoSlo‐SR compounds. In addition, except in the tail artery, GoSlo‐SR‐induced vasodilation was reduced by inhibition of K_v_7 channels either partly, when BK channels were not blocked, or fully when BK channels were blocked.

A possible explanation for our observations has been suggested recently (Coleman, Tare, & Parkington, 2017). If the GoSlo compounds produce a considerable hyperpolarisation, the membrane potential will be much closer to the potassium equilibrium potential resulting in a small driving force for potassium ions. Due to the small driving force, blockade of potassium channels under these conditions will result in only a small change in membrane potential and vessel tension. Thus, our data are consistent with the idea that when IBTX has blocked functional BK channels at small driving force, membrane potential was almost not affected, and the effect of GoSlo was unchanged. When XE991 has blocked functional K_v_7 channels (with a somewhat larger impact, compared to blocking BK channels) under conditions where the driving force is small, membrane potential was presumably affected and the effect of GoSlo was reduced. However, when IBTX together with XE991 has blocked functional BK and K_v_7 channels, the effects on GoSlo on membrane potential were presumably blocked, and as a result, the GoSlo‐induced relaxation was attenuated.

In conclusion, the data presented in this study show that GoSlo compounds are much more effective relaxants in vascular compared to visceral smooth muscle. Like other small molecule BK channel openers, GoSlo‐SR compounds mediate their vasodilator effects by a combined activation of BK and K_v_7.4/K_v_7.5 channels. Activation of K_v_1, K_v_2, or K_v_7.1 channels or other vasodilator pathways, for example, voltage‐gated calcium channels, seems not to be involved in this effect. Whereas the joint activation of BK and K_v_7.4/K_v_7.5 channels by the GoSlo‐SR compounds is not without precedent, the GoSlo‐SR compound‐induced vasodilation was characterised by a special feature. This vasodilation was mediated by K_v_7.4/7.5 channels only when BK and K_v_7.4/7.5 channels were available but was mediated by BK channels when K_v_7.4/7.5 channels were not available. This special mechanism of action of GoSlo‐SR compounds may be beneficial for their clinical use as K^+^ channels openers, for example, against combined BK and K_v_7 channel dysfunction like in hypertension. This idea has to be confirmed in future studies.

## CONFLICT OF INTEREST

M.A.H., G.P.S., and K.D.T. hold a patent on the GoSlo family of compounds (USPTO 9877940).

## AUTHOR CONTRIBUTIONS

M.A.H. and R.S. designed the study; O.Z., S.D., D.M., R.K., S.A., D.T., M.K., K.T., M.M., C.K., G.S., N.M., O.W., and H.W. performed the experiments; O.Z., S.D., D.M., R.K., S.A., D.T., M.K., K.T., M.M., G.S., A.K., M.G., M.A.H., and R.S. analysed the data; N.M. synthesised and purified the GoSlo compounds; M.A.H. and R.S. wrote the manuscript; O.Z., S.D., D.M., R.K., S.A., D.T., M.K., K.T., M.M., C.K., G.S., N.M., O.W., H.W., A.K., M.G., M.A.H., and R.S. have read and approved the manuscript.

## DECLARATION OF TRANSPARENCY AND SCIENTIFIC RIGOUR

This Declaration acknowledges that this paper adheres to the principles for transparent reporting and scientific rigour of preclinical research as stated in the *BJP* guidelines for https://bpspubs.onlinelibrary.wiley.com/doi/full/10.1111/bph.14207, and https://bpspubs.onlinelibrary.wiley.com/doi/full/10.1111/bph.14206, and as recommended by funding agencies, publishers and other organisations engaged with supporting research.
